# Generation and analysis of a barcode-tagged insertion mutant library in the fission yeast *Schizosaccharomyces pombe*

**DOI:** 10.1186/1471-2164-13-161

**Published:** 2012-05-03

**Authors:** Bo-Ruei Chen, Devin C Hale, Peter J Ciolek, Kurt W Runge

**Affiliations:** 1 Department of Genetics, Case Western Reserve University School of Medicine, 10900 Euclid Avenue, Cleveland, OH, 44106, USA; 2 Department of Molecular Genetics, Cleveland Clinic Lerner College of Medicine at CWRU, 9500 Euclid Avenue, NE20, Cleveland, OH, 44195, USA; 3 John Carroll University, 20700 North Park Boulevard, University Heights, Ohio, 44118, USA; 4 Miami University, 501 East High Street, Oxford, OH, 45056, USA; 5 Current address: West Virginia School of Osteopathic Medicine, 400 North Lee Street, Lewisburg, WV, 24901, USA; 6 Current address: College of Medicine, Ohio State University, 370 West 9th Avenue, Columbus, OH, 43210, USA

## Abstract

**Background:**

Barcodes are unique DNA sequence tags that can be used to specifically label individual mutants. The barcode-tagged open reading frame (ORF) haploid deletion mutant collections in the budding yeast *Saccharomyces cerevisiae* and the fission yeast *Schizosaccharomyces pombe* allow for high-throughput mutant phenotyping because the relative growth of mutants in a population can be determined by monitoring the proportions of their associated barcodes. While these mutant collections have greatly facilitated genome-wide studies, mutations in essential genes are not present, and the roles of these genes are not as easily studied. To further support genome-scale research in *S. pombe*, we generated a barcode-tagged fission yeast insertion mutant library that has the potential of generating viable mutations in both essential and non-essential genes and can be easily analyzed using standard molecular biological techniques.

**Results:**

An insertion vector containing a selectable *ura4*^+^ marker and a random barcode was used to generate a collection of 10,000 fission yeast insertion mutants stored individually in 384-well plates and as six pools of mixed mutants. Individual barcodes are flanked by *Sfi* I recognition sites and can be oligomerized in a unique orientation to facilitate barcode sequencing. Independent genetic screens on a subset of mutants suggest that this library contains a diverse collection of single insertion mutations. We present several approaches to determine insertion sites.

**Conclusions:**

This collection of *S. pombe* barcode-tagged insertion mutants is well-suited for genome-wide studies. Because insertion mutations may eliminate, reduce or alter the function of essential and non-essential genes, this library will contain strains with a wide range of phenotypes that can be assayed by their associated barcodes. The design of the barcodes in this library allows for barcode sequencing using next generation or standard benchtop cloning approaches.

## Background

Current genome-wide analyses mainly depend on either gene expression profiling or large-scale mutant phenotyping (e.g. [1,2]). Expression profiling allows for the detection of changes in gene expression levels; however, the pattern of gene expression does not always reflect gene function. For example, in a genome-wide analysis of the budding yeast *Saccharomyces cerevisiae* gene deletion mutant set, less than 7% of the genes that showed increased mRNA expression in response to four different conditions were required for growth under the same conditions, and deletion of some of the most highly expressed genes had no effects on cell proliferation. In addition, many genes necessary to maintain normal cell fitness under these treatments did not have significantly altered expression levels [2].

Large-scale mutant phenotyping monitors changes in mutant fitness or other visible traits, and provides direct assessment of the requirements of genes under specific conditions. The availability of the open reading frame (ORF) deletion mutant collections in the budding yeast *S. cerevisiae* and the fission yeast *Schizosaccharomyces pombe* has proven to be advantageous in this approach. The principal challenge in large-scale phenotyping is distinguishing individual mutants. The current method of choice is to tag each mutation with a unique DNA sequence called a “barcode” [3,4]. Because barcode tags are part of the mutations, the proportion of an individual barcode reflects the proportion of that barcode-associated mutant in a population. Thus, following barcode frequencies can identify mutants with a desired growth phenotype from a population of diverse mutants, an approach referred to as parallel analysis [3,4].

An advantage of parallel analysis of barcoded mutants is that mutations causing deleterious or weak phenotypes can be efficiently detected. For example, to identify virulence genes in *Salmonella typhimurium*, mice were infected with a *S. typhimurium* mutant library where each mutant carried a unique 40 bp barcode tag. The barcodes that were lost from the post-infection population identified the genes required for virulence [3]. This approach has also been extended to cultured human cells using barcode-tagged cDNA and shRNA libraries to discover genes whose overexpression and down-regulation could suppress cancer cell growth and survival [5,6]. In all three screens, the mutants of interest diminished in the population but could be revealed by detecting the loss of their associated barcodes by DNA arrays [3,5,6].

The budding yeast *S. cerevisiae* is the model organism where barcode-tagged mutagenesis has been the most successfully exploited [2,4,7]. Its small, sequenced and well-annotated genome and the efficient gene deletion techniques allowed the production of a collection of complete ORF deletion mutants where each deletion mutant is tagged by two unique barcodes [2]. The barcodes can be amplified *en masse* by PCR to generate probes for commercially available high-density microarrays to take a “census” of the relative abundance of each mutant in a culture under a variety of conditions [2]. Two independent genetic screens using this barcode-tagged deletion mutant set have identified many genes whose deletion caused lengthened chronological lifespan as detected by an increase in the abundance of the long-lived mutant barcodes [8,9]. As long-lived mutants and mutants with normal lifespan are often morphologically indistinguishable, the barcode approach to monitor the length of lifespan of many mutants in parallel demonstrated the power of parallel analysis for detecting weak phenotypes.

*S. pombe* is an important model system that has many of the advantages of *S. cerevisiae*, including a sequenced genome and well-established microbiological, genetic and molecular biological approaches [10,11]. Unlike *S. cerevisiaeS. pombe* shares a number of similar features with mammals including RNA interference, aspects of RNA splicing and the requirement for the mitochondrial genome for survival of wild type cells [12-16]. A barcode-tagged deletion strain set for *S. pombe*, in which each deletion mutant carries two barcodes, has also been developed recently [17].

Most of the mutants in the *S. pombe* and *S. cerevisiae* ORF deletion sets are null mutations, and haploid mutants lacking essential genes are not present. Truncated essential proteins with partial or altered function and mutated alleles of essential genes with reduced expression levels have been shown to produce viable and detectable phenotypes such as changes in transcriptional silencing and lifespan [18,19]. Therefore, a mutant collection generated by a different approach that includes mutations that impair the function of essential and non-essential genes would be beneficial for parallel analysis of phenotypes and genome-wide screens.

An alternative mutagenesis method in *S. pombe* is non-homologous recombination-dependent integration of a selectable marker [20,21], which can generate a wide variety of mutations. Insertions in the coding sequences can produce truncated proteins with either no or altered function. Insertions in the 5’ and 3’ region of a gene can change protein expression levels by compromising promoter function and mRNA stability, respectively [18,22,23], and produce viable mutations in essential genes [18]. Previous studies in *S. pombe* characterized two classes of transformants when cells were transformed with linear DNA that had limited or no homology to the genome [24,25]. One class of transformants contained single or tandem copies of linear DNA stably inserted in the genome, and the insertion events were often accompanied by deletions in the integrated DNA vectors and adjacent genome sequences. The distribution of insertions in the genome appeared random [24,25]. The other class, which constituted the majority of the transformants, maintained the transfected DNA as unstable, extrachromosomal circular DNA despite the absence of a known origin of DNA replication in the vector [20,21,24-26].

Chua et al. [20] and Davidson et al. [21] used this approach to create random *S. pombe* mutants by transforming a ~1.7 kb DNA fragment containing the *ura4*^+^ gene into strains with the *ura4-D18* mutation, a deletion that removes the sequences homologous to the 1.7 kb *ura4*^+^ DNA from the genome. These studies revealed two important features. First, only one insertion event was identified in each mutant by the criteria of Southern blotting. Thus, it is possible to generate a library of transformants where each individual contains a single mutation [20,21]. Second, many of the transformants contained insertions composed of multiple full or partial copies of the vector at each site. The complex structure of these insertions made mapping their genomic locations challenging [21].

To facilitate genome-wide functional analysis in *S. pombe*, we generated a barcode-tagged *S. pombe* library of random insertion mutants that retains features equivalent to the budding and fission yeast ORF deletion mutant collections, enables identification of mutants in the absence of knowing the barcode sequences, and allows parallel analysis with basic molecular biology techniques. We created an insertion DNA cassette composed of the *ura4*^+^ selectable marker, a random barcode, a “buffer” sequence to protect the barcode from degradation during integration, and other sequences to allow modifications of the integrated vector. Of the ~10,000 insertion mutations generated, phenotypic analysis and mapping of a subset indicated that the insertions were broadly distributed in the genome. Thus, this work demonstrates the feasibility and potential utility of constructing barcoded, random insertion libraries in *S. pombe*, and provides approaches that can allow rapid analysis of large collections of barcode-tagged mutations in other organisms.

## Results

### A linear DNA vector loses sequences from the ends during non-homologous recombination-mediated insertion in *S. pombe*

**Figure 1:**
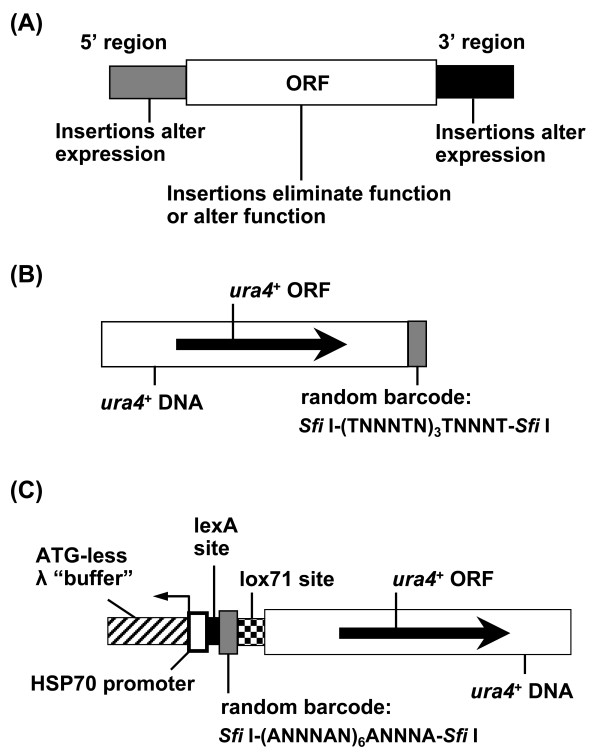
**A non-homologous recombination-mediated insertion mutagenesis for generating an*S. pombe*mutant library.** (**A**) The first insertion vector tested had the selectable marker *ura4*^+^ and a 15-bp random barcode directly following the 3’ UTR of *ura4*^+^. (**B**) The insertion vector used to construct the *S. pombe* insertion mutant library is composed of a selectable marker *ura4*^+^ gene, a barcode (27 random nucleotides with 14 interspersed A’s), a lox71 site for one-way integration of lox66-bearing DNA, a mutated human HSP70 promoter with a lexA binding site and a modified λ phage sequence, ATG-less λ, to protect the sequences 3’ to the λ phage fragment from degradation.

As a first test of using insertion mutagenesis to generate barcode-tagged insertion mutations in *S. pombe*, an initial barcoded insertion vector was made by PCR amplification of the *ura4*^+^ gene using *ura4*^+^-specific primers to produce a *ura4*^+^ gene with a random barcode sequence at its 3’ end (Figure 1A). Cells with the *ura4-D18* mutation were transformed with the linear vector DNA and maintained on minimum medium without uracil to select for Ura^+^ transformants. Anticipating that the majority of the transformants would carry extrachromosomal *ura4*^+^ circles, the initial transformants were re-grown twice on non-selective medium to allow cells to lose unstable *ura4*^+^ circles. These colonies were then replica-plated onto both selective medium and minimum medium plates with 5-fluoroorotic acid (5-FOA, 1 g/l) to identify transformants with stably integrated *ura4*^+^, which are inviable in 5-FOA medium [27,28].

The initial test with 199 Ura^+^ transformants identified eight that were sensitive to 5-FOA, indicating that only about 4% of the transformed cells contained stably integrated DNA. The rest of the transformants (96%) likely carried the *ura4*^+^ marker extrachromosomally and had lost the circular *ura4*^+^ DNA upon growth on 5-FOA-containing medium. Analysis of the eight *ura4*^+^ insertion mutants by PCR revealed that seven mutants had lost their barcodes, indicating that successful integration was frequently accompanied by deletion of barcodes. These results are consistent with previous observations of low frequency stable integration by non-homologous recombination and deletion on the ends of the inserted DNA [20,21,25], underscoring the need to increase the proportion of stable transformants and to prevent deletion of barcodes.

### Construction and characterization of the bacterial barcode-tagged insertion DNA library

The final insertion DNA vector contains a barcode between a “buffer sequence” and the 5’ end of the *ura4*^*+*^ gene so deletions would first occur in the buffer sequence or the selectable marker before altering the barcode (Figure 1B and Table 1). This buffer sequence included new sequence elements with additional potential functions: a modified 250 bp λ DNA sequence (ATG-less λ) at the 5’ end to prevent the degradation of the barcode, followed by a modified human HSP70 promoter that contains a lexA binding site [29,30], and a mutated loxP site named lox71 [31]. The lexA-HSP70 promoter can be bound by any lexA fusion protein and may stimulate transcription through the λ DNA sequences, which have been modified to contain no ATG sequences, and would translate the first ATG in *S. pombe* genomic DNA [32]. The lox71 site can, in the presence of Cre recombinase, recombine with the lox66 site to allow integration (but not excision) of a plasmid [31].

**Table 1:** Summary of individual components of the insertion vector

**DNA sequence**	**Function**
ATG-less λ buffer	Protect the lexA-HSP70 promoter and the barcode from degradation
HSP70 promoter	Drive the expression of adjacent genes
lexA site	The binding site for LexA-VP16 protein for inducible activation of the HSP70 promoter
Random barcode	A specific DNA tag in each mutant for tracking mutant frequency
*ura4*^+^ ORF/DNA	The selectable marker

**Figure 2:**
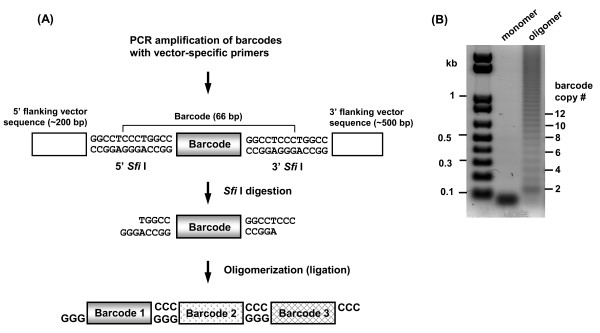
**Ligation-mediated barcode oligomerization.** (**A**) Barcodes in this insertion mutant library can be amplified from a population of mutants by PCR using vector-specific primers that flank barcodes. Overhangs generated by *Sfi* I digestion allow barcode monomers to be oligomerized in a head-to-tail manner. (**B**) A representative barcode oligomerization. The lanes labeled “monomer” and “oligomers” show the *Sfi* I-digested barcode DNA before and after ligation, respectively.

The new barcode consists of a 27-nucleotide random sequence that is interrupted at specific positions with 14 A bases. This “interrupted barcode” is flanked by two *Sfi* I restriction sites so that the barcodes can amplified by PCR, digested with *Sfi* I restriction endonuclease and oligomerized in a head-to-tail orientation for sequencing several barcodes in a single reaction (Figure 2A). As a test of this approach, a fragment containing the barcode DNA and flanking insertion vector sequences was first PCR amplified and then digested with *Sfi* I. Gel-purified barcode monomers were then ligated together to form oligomers (Figure 2B, details in Materials and Methods). A ladder of barcode oligomers was generated, and cloning and sequencing of those oligomers longer than five barcodes confirmed that they were ligated in a head-to-tail manner. We have successfully cloned and sequenced up to 16 tandem barcodes per plasmid, and could routinely clone an average of seven barcodes per plasmid in pilot experiments (data not shown). Thus, when sampling a population of random mutants, each sequenced plasmid provides information on about seven different barcodes and sequencing 20 to 30 plasmids can provide a sufficiently large sample size to identify barcodes that are present at high frequency.

As the barcodes used in this work were designed with 27 random bases, they provide a total of 4^27^ or 1.8 × 10^16^ possible barcode sequences. We generated a barcode library of 6.99 × 10^6^ bacterial clones from 37 sub-libraries containing 8.5 × 10^4^ to 2.6 × 10^5^ clones per sub-library. Each sub-library contains independent and different random barcodes and helps preserve the high complexity of the library. Our goal was to generate 10,000 fission yeast mutants with unique and non-redundant barcodes, which should give a 76% chance to cover all annotated genes (details in Materials and Methods). In general, the number of fission yeast mutants produced was ≤ 1% of the total number of barcode clones present in the bacterial sub-libraries used for mutagenesis, so all mutants have a ≥ 95% chance of having unique barcodes.

### Construction of the fission yeast barcode-tagged insertion mutant library

The 2.1 kb linear barcoded insertion vector DNA was purified from the bacterial barcode sub-libraries after digestion with *BamH* I enzyme and used to generate the initial transformants. To enrich the overall proportion of stable integrants, we tested the possibility of utilizing low concentrations of 5-FOA to select against cells bearing multiple copies of extrachromosomal *ura4*^+^ circles. Cells bearing *ura4*^*+*^ circles are expected to have multiple *ura4*^*+*^ genes per cell. Higher levels of Ura4 protein should produce more toxic 5-fluorouracil in the presence of low concentrations of 5-FOA, causing these cells to have a growth disadvantage (Additional file 1, Figure S1A). In contrast, stable integrants with one or few copies of integrated *ura4*^*+*^ DNA do not produce as much Ura4 protein, and are less sensitive to the same treatment (Additional file 1, Figure S1B). To test this assumption, fission yeast cells transformed with the linear insertion vector DNA were immediately plated on low-dose 5-FOA selective medium plates (100 mg/l of 5-FOA, the highest concentration in which the cells with a single copy of *ura4*^+^ gene can survive; unpublished observation). The resulting transformants were verified for stable integration by growing them on normal-dose 5-FOA medium (1 g/l of 5-FOA) and minimum medium plates without uracil. An initial test on 100 transformants grown on low-dose 5-FOA medium plates identified 30 transformants which died when transferred to medium with normal-dose 5-FOA plates. This result indicates 30% of the transformants contained stably inserted vector DNA, a significant enrichment of mutants with stable integration compared to the previously observed 4%.

**Figure 3:**
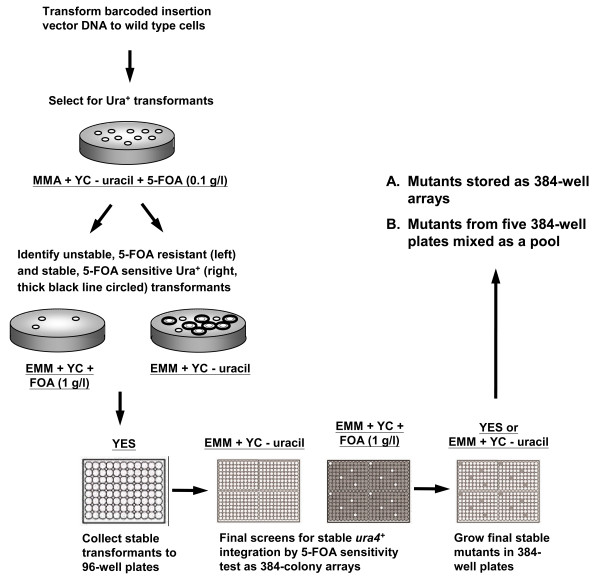
**Generation of the barcode-tagged*S. pombe*insertion mutant library.** The linear insertion DNA (Figure 1B) was transformed into the wild type strain KRP1 to obtain Ura^+^ transformants on minimal medium (MMA) with multiple nutritional supplements except uracil (YC – uracil) and low levels of 5-FOA (0.1 g/l). Transformants were then tested for stable integration by 5-FOA sensitivity. Stable transformants (i.e. 5-FOA sensitive cells) were inoculated in non-selective YES medium in 96-well plates, followed by assembling four such plates on a synthetic medium plate lacking uracil (EMM + YC – uracil) and a similar medium plate that contains uracil and 1 g/l of 5-FOA (EMM + YC + 5-FOA) to generate 384-colony arrays for the second 5-FOA sensitivity test. Unstable transformants found in this second screen were removed before these mutants were stored as 384-well mutant arrays or mixed mutant pools of ~1800 mutants.

The low-dose 5-FOA selection procedure was used in large-scale generation of fission yeast insertion mutants (Figure 3). Transformants obtained on low-dose 5-FOA selective medium plates and passing the first verification on normal-dose 5-FOA medium plates were transferred to non-selective medium in 96-well plates. Each four 96-well plates were then used to make one 384-colony array on a selective medium and a normal-dose 5-FOA Omni plate to confirm the Ura^+^ and 5-FOA-sensitive phenotypes of stable insertion mutants. Mutants behaving as true stable insertion mutants were stored individually in 384-well plates (28 plates in total). In addition, five ~1,800-mutant pools (each from five 384-well plates) and one ~1100 mutant pool (from three 384-well plates) were made for a total of ~10,000 mutants.

### The insertion mutant library contains diverse mutations

To evaluate the diversity of mutations in the insertion mutant library, four genetic screens were performed on 3600 mutants from ten 384-well plates for phenotypes that could be easily scored.

In the first screen, we looked for mutants that grew slowly or could not grow on minimum medium plates supplemented with only adenine, leucine and histidine to complement the original auxotrophic mutations in our strain. We discovered 30 mutants containing mutations that impeded growth enough to prevent visible colony formation on minimal medium. Based on the Gene Ontology database (GO)-predicted mutant frequency [33] and the fact that only ~57% of the *S. pombe* genome contains protein coding genes in which mutations are more likely to generate detectable phenotypes than in intergenic regions [10], 37 auxotrophic mutants would be expected in the 3600 insertion mutants assayed (Table 2). Because not every mutation will generate a visible phenotype, the frequency of auxotrophic mutants in this assay was consistent with the GO prediction.

**Table 2:** Assessment of mutation diversity in the barcode-tagged insertion mutant library by four genetic screens

**Mutation**	**Phenotype**	**AmiGO or** **KEGG expectation**	**Expected number in 3581 mutants**	**Actual number of isolates**
Auxotrophy	Slow or no growth on minimal medium	2.4% (94^a^/5122^b^)	37^c^	30
Defective adenine biosynthesis	Colony color change from pale pink to white or red on low adenine medium	ND^d^	ND^d^	13 (white^d^)
		0.04% (2^e^/5122 ^b^)	1-2^e^	1 (red)
Temperature sensitivity	Slow or no growth at 36°C	ND^f^	ND^f^	25
EtBr resistance (petite positivity)	Growth in medium with EtBr	ND^f^	ND^f^	13

a. The estimated number is from AmiGO database [33] by searching genes using keywords “amino acid biosynthesis” and “nucleobase biosynthesis”, and excluding genes involved in the adenine, histidine, leucine and uracil pathways as the parental strain in defective in these pathways and all mutants are Ura^+^.

b. Total gene number in *S. pombe* genome (as of 2/27/2012) = 5122.

c. The estimated number is calculated as “the total number of mutants tested (3581)” × “AmiGO expectation” × “the fraction of protein coding sequences in the total *S. pombe* genome (57%)”.

d. Not determinable; white colony color could also result from mitochondrial defects [34,35].

e. The estimated number is from KEGG [36] (*ade6*^*+*^ and *ade7*^*+*^).

f. Not determinable; no associated terms or categories in AmiGO or KEGG.

In the second assay, we selected for mutations that altered colony color on low adenine medium. As the parental yeast strain of the insertion library mutants contains the *ade6-M216* mutation, we screened for an alteration in colony color from pale pink (the *ade6-M216* phenotype) to red or white when cells were grown in rich medium with low concentrations of adenine. Mutations in only two genes, *ade6*^+^ and *ade7*^+^, are known to cause red pigment accumulation in cells. One mutant out of the 3600 screened showed dark red colony color on low adenine medium plate and was identified as an *ade7*^*-*^ insertion mutant (Table 2 and mutant 13_C10 in Additional file 2: Table S1). Thirteen mutants which turned white on low adenine medium plates were also isolated. Although this phenotype can be associated with mutations in the adenine biosynthesis pathway that produce substrates for the Ade6 and Ade7 enzymes, mutants with compromised mitochondrial function have also been reported to demonstrate a similar phenotype [34,35].

In the third test, we screened for temperature-sensitive mutations that allow mutant cells to grow normally at 30°C, but not at 36°C. A total of 25 such mutants that grew slowly or could not form visible colonies at 36°C were recovered (Table 2). Mutations that cause temperature-sensitive growth are not well-characterized, so these data cannot be used to estimate the expected mutant frequency. However, the isolation of temperature-sensitive mutants does demonstrate the wide variety of mutations present in the library.

In the fourth experiment, we selected for mutations that confer cellular resistance to ethidium bromide (EtBr). EtBr toxicity is primarily due to inhibition of the circular mitochondrial genome. As with mammalian cells, *S. pombe* requires a functional mitochondrial genome for survival so EtBr is highly toxic. If *S. pombe* and mammalian cells acquire certain nuclear mutations, the so-called *rho*^0^ cells devoid of mitochondrial DNA can be generated by long term selection in EtBr-containing medium with specific supplements [12,37]. Using this EtBr selection procedure, we identified 13 strains that are EtBr-resistant (Table 2).

To determine whether these mutant phenotypes were caused by the *ura4*^+^ insertion or mutations induced by transformation, we crossed the mutants to a wild type strain and performed tetrad analysis on a subset of the identified mutants to verify co-segregation of the *ura4*^+^ marker and the phenotypes. Of 34 strains examined, 28 showed 2:2 segregation of *ura4*^+^ in (on average) seven tetrads (Additional file 2: Table S1). By this criteria, the majority of mutants contained single insertions. Of the 18 mutants that showed slow or no growth on minimum medium, the *ura4*^+^ marker co-segregated with the phenotype in a 2:2 ratio in 16 mutants, indicating that most of the mutant phenotypes were linked to the insertion mutations. Similar results were observed in mutants exhibiting altered color on low adenine medium (six out of 10 mutants show complete co-segregation of phenotypes and markers, Additional file 2: Table S1). Of the 11 temperature-sensitive mutant assayed, one was sterile, four showed poor spore viability and three of the remaining six showed 2:2 co-segregation with *ura4*^+^ (Additional file 2: Table S1 and data not shown). The source of the unlinked mutations is unknown but may be due to the mutagenic effects of transformation [38,39], and underscores the requirement of validating mutants from premade collections by recreating the mutation in a new strain to determine if the phenotype is regenerated. The sum of these data show that the majority of these insertions contain the *ura4*^+^ vector in a single locus and the insertion locus is genetically linked to the mutation in most of these mutants.

### Mapping of insertion sites and analysis of the structures of inserted vectors

Previous studies on non-homologous recombination-mediated mutagenesis in *S. pombe* showed that the genomic locations of insertion vectors were difficult to determine by inverse PCR [21]. We therefore tested two previously established methods for large-scale insertion site determination, thermal asymmetric interlaced (TAIL)-PCR [40,41] and splinkerette-adaptor PCR [42,43]. TAIL-PCR uses alternating high and low annealing temperatures, a set of arbitrary degenerate (AD) primers and three nested insertion DNA-specific primers to amplify a small region of insertion DNA and the adjacent genomic sequence (Additional file 3: Figure S2A) [40,41]. TAIL-PCR only detected genome-insertion junctions in a portion of mutants analyzed (described below). In some mutants, only repetitive insertion vector sequences were amplified, indicative of tandem integration of vector DNA in these mutants (data not shown). In other mutants, mitochondrial DNA was found co-integrated with insertion vector (data not show). These results indicate that these additional DNA fragments provided binding sequences for the degenerate or vector-specific primers (Additional file 3: Figure S2B, C). Therefore, we also used splinkerette PCR, which involves ligating a double strand DNA adaptor to genomic DNA fragments digested with restriction enzymes that cut frequently in the genome but not in the vector (e.g. *Spe* I and *Xba* I, Additional file 4: Figure S3) [44]. The resulting products provide templates for PCR amplification of neighboring genomic DNA using specific primers on the splinkerette adaptor and insertion vector. The splinkerette approach produced PCR fragments of defined size and some genomic sequences, allowing the estimation of the region of the insertion in some mutants and detection of insertion-chromosome junction in others (Additional file 2: Table S1). As the splinkerette approach also gave vector or mitochondrial sequences in some mutants, we pursued additional mapping approaches.

**Figure 4:**
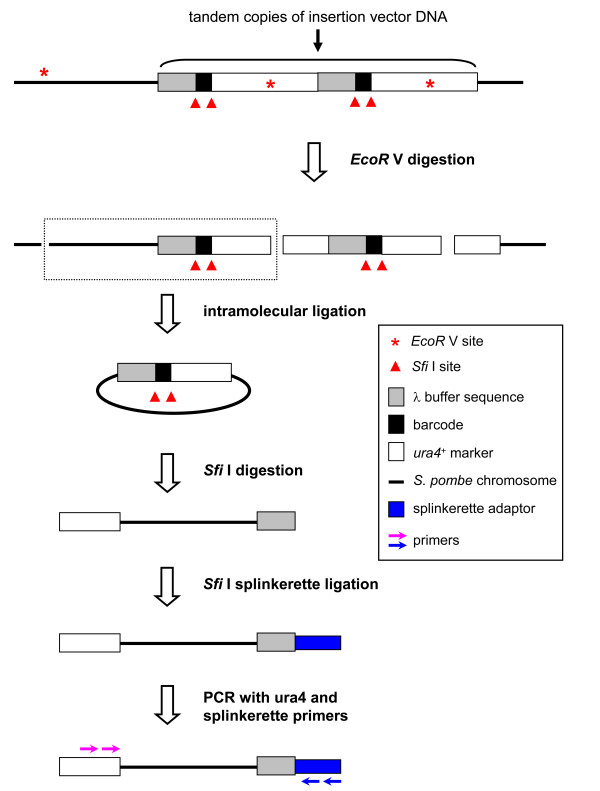
**Inverse splinkerette PCR.** Genomic DNA of an insertion mutant is first digested with a restriction enzyme that cuts once (or very few times) in the insertion vector and frequently in the genome (*EcoR* V). The resulting products are ligated to generate DNA circles. Digestion with *Sfi* I produces one end with partially degraded vector DNA (λ buffer) for ligation of a double-strand splinkerette adaptor. Genomic DNA bordered by the splinkerette and the partial *ura4*^+^ marker can be amplified by nested splinkerette and *ura4*^+^ primer sets in two rounds of PCR.

We adapted splinkerette and inverse PCR to a new method, inverse splinkerette PCR, which may eliminate extra copies of tandem insertion vector in some mutants before PCR (Figure 4). In inverse splinkerette PCR, genomic DNA from insertion mutants was digested with a restriction enzyme that cuts once (or very few times) in the insertion vector and frequently in the genome (e.g. *EcoR* V in this work). The digestion generates fragments with one chromosomal DNA end and one insertion vector end, which can be brought together by intramolecular ligation to produce circular DNA. Digestion of the ligated DNA with *Sfi* I enzyme linearizes circular DNA composed of truncated 5’ part of the insertion vector and a genomic fragment, and generates a unique overhang on the λ buffer of vector for ligating splinkerette adaptor. The genomic DNA bordered by the partial vector DNA and splinkerette adaptor can be amplified by specific primers on insertion vector and splinkerette. This method can reveal the *EcoR* V site closest to the insertion site and together with the length of the PCR product, to generate an approximate location of the insertion mutation (Additional file 2: Table S1). In cases where the *EcoR* V site is very close to the insertion site, one may detect the precise junction sequences between chromosomes and insertion vector.

**Figure 5:**
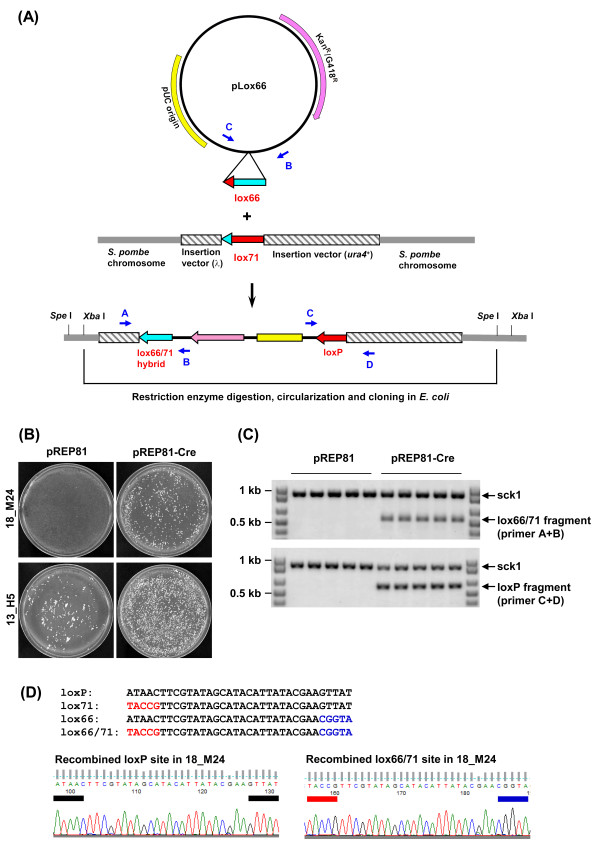
**Lox66/lox71-mediated DNA integration and cloning of insertion mutation.(A)** Integration of a bacterial plasmid DNA into the insertion mutation of a *S. pombe* insertion mutant. The plasmid pLox66 bearing the lox66 sequence can recombine with lox71 on the integrated insertion vector in *S. pombe* in the presence of Cre recombinase. After pLox66 integration, pLox66, the insertion vector and nearby *S. pombe* genomic DNA can be excised by restriction digestion and cloned in *E. coli*. The pUC origin allows pLox66 to be amplified and maintained in *E. coli* and Kan^R^/G418^R^ gene (*kanMX*) allow selection of the plasmid in *E. coli* (kanamycin resistance) and *S. pombe* (G418 resistance). **(B)** Cre recombinase-dependent integration of pLox66 in *S. pombe*. The pLox66 DNA was transformed to *S. pombe* insertion mutant strains 18_M24 or that expressed or did not express Cre recombinase (pREP81-Cre or pREP81). Transformed cells were replica plated to solid media with G418 to test G418 resistance and stable integration of pLox66. **(C)** Stable integration of pLox66 in 18_M24 was tested by PCR using primers on the insertion vector (A and D in panel A) and pLox66 (B and C in panel A). A truncated *sck1*^+^ gene fragment was co-amplified in each reaction as a positive control. Five independent colonies of each transformation were tested. **(D)** PCR products of 18_M24 with pREP81-Cre were sequenced to examine the recombined wild type loxP and lox66/71 hybrid sequences. The colored boxes in the electropherograms highlight the base differences in the individual lox71 and lox66 sites while the black boxes indicate the wild type loxP sequences.

We also directly cloned the genomic sequences flanking the insertion vector in *E. coli* by the lox66/lox71 integration system (Figure 5A). As the insertion mutations are all marked with the mutated loxP sequence, lox71, introducing a plasmid bearing the lox66 sequence into cells expressing Cre recombinase should allow stable integration of the lox66 plasmid into the lox71 sequence. We therefore transformed the plasmid pLox66, which has the lox66 sequence and a selectable marker for G418 resistance in yeast and kanamycin resistance in *E. coli*, into *S. pombe* insertion mutant cells bearing the pREP81 plasmid with or without the Cre recombinase gene. Because pLox66 does not contain a functional yeast replication origin, integration into the genome is required for its stable inheritance and G418 resistance in yeast cells. Following introduction of pLox66, cells expressing Cre recombinase produced more G418-resistant colonies than cells without Cre, indicating that Cre promotes efficient integration of pLox66 into the genome (Figure 5B). Specific integration of pLox66 to lox71 of the insertion vector was verified by PCR using primers on pLox66 (B, C, Figure 5A) and the insertion vector (A, D, Figure 5A). While expected PCR products were generated from Cre-expressing cells, the same PCR reactions using those G418-resistant cells without Cre did not yield bands corresponding to pLox66 integration, indicating these cells may bear pLox66 extrachromosomally (Figure 5C). Sequencing of PCR products from cells expressing Cre also confirmed that they contained the recombined lox66/lox71 hybrid and the wild type loxP sequences (Figure 5D).

To clone genomic DNA flanking the insertion sites, genomic DNA of G418-resistant, pLox66-integrated cells was digested with *Spe* I and *Xba* I, and ligated under conditions favoring intramolecular ligation. The presence of the pUC origin on pLox66 allowed us to clone genomic sequences neighboring the insertion site in *E. coli* for sequencing (Figure 5A). Using this method, we cloned and verified the insertion site of the mutant 18_M24 (Additional file 2: Table S1).

Using TAIL-PCR, we initially characterized 44 mutants, of which we were able to determine the genomic sequences at one end of the insertion sites in 24 mutants. In 12 mutants, only repetitive insertion vector sequences were amplified and sequenced, and mitochondrial DNA was found co-integrated with insertion vector in eight mutants (Additional file 2: Table S1 and data not shown). We have also used inverse splinkerette PCR to map insertions in 38 mutants and determined approximate location of the insertion vector in 14 mutants, nine of which we were not able to determine by TAIL-PCR (Additional file 2: Table S1). These results suggest that although TAIL-PCR and inverse splinkerette PCR are not sufficient for mapping all insertion sites, they are complimentary to each other in deciphering insertion positions accompanied by different insertion structures. Splinkerette PCR and lox66/71-based cloning also allowed the determination of insertion sites (Additional file 2: Table S1).

Among the 38 mutants in which exact or approximate insertion locations were determined, 13 of them have insertion mutation on chromosome 1, with 16 on chromosome 2 and 9 on chromosome 3. In the 27 mutants where at least one end was mapped to a defined locus, 16 were located in ORFs or non-coding RNA genes and 11 were found in intergenic regions.

In the seven mutants in which both ends of the insertion sites were characterized, deletions of genomic sequences (≤ 5 bp) were found in only three mutants (13_H5, 18_M24 and 1a7-4033, Additional file 5: Table S2), and deletions of a wide range of sizes, from 5 bp to 1.8 kb, were observed at ends of the insertion vectors in all mutants. Tandem integration of multiple insertion vectors were detected in all but one mutant (1a-4032) in head-to-tail, head-to-head or tail-to-tail orientations. The structures of the insertion vectors in these mutants is consistent with the ends of some linear vector DNA being resected by nucleolytic activities in cells and ligated together before being integrated as tandem copies into the genome as previously observed by others (Additional file 5: Table S2 and Additional file 6: Figure S4, and [20,21,25]).

## Discussion

In this study, we created a fission yeast insertion mutant library in which all mutants were tagged with unique barcode sequences and stored as two readily available selection platforms. The 384-well mutant arrays allow genetic screens on individual mutants and can be extended to genetic approaches such as synthetic genetic array (SGA) [45,46]. These mutant arrays have been used to identify mutants with four distinct phenotypes (Table 2) as well as strains that are hyper-sensitive to cancer chemotherapeutics camptothecin and bleomycin (Hale and Runge, unpublished data). In addition to 384-well mutant arrays, mutant pools of 1800 mutants are available for parallel analysis.

The insertion mutagenesis used in this study relied on random non-homologous recombination, where a vast majority of transformants have unstable, circularized vector DNA and only a small portion have stable insertions. To facilitate the collection of stable insertion mutants, we included low-dose 5-FOA in our initial selecting medium as an effort to eliminate unstable cells bearing high copy number of *ura4*^+^ vector and producing high levels of Ura4p (Additional file 1: Figure S1), and subsequently re-screened for mutants that were stably *ura4*^+^ and 5-FOA-sensitive (Figure 3). While this approach increased the proportion of stable insertion mutants among the total transformants (from 4% to 30%), we note that some mutants might have been excluded. For example, insertions into genomic regions where expression switches between on and off states (e.g. telomeres [47]) would be excluded from the final library. Likewise, insertion at a locus that causes high *ura4*^+^ expression or tandem integration of many functional *ura4*^+^ markers could result in increased sensitivity to low-dose 5-FOA and eliminate some mutants during the initial selection.

To increase the versatility of the mutant library, some previously characterized functional DNA sequences were included in our insertion vector, including the lox71 sequence and the mutated human HSP70 promoter. We demonstrated that the mutated lox66 and lox71 could undergo Cre recombinase-dependent integration in *S. pombe*, similar to what has been reported in mammalian cells [29], and showed that this method can be used to clone genomic sequences surrounding the insertion mutations using the pLox66 plasmid. The mutated human HSP70 promoter, which exhibits dramatically reduced activity in *S. pombe*, was tethered with a lexA binding site for its potential activation by a lexA DNA-binding domain and transactivator fusion protein. While the goal was to provide an opportunity to ectopically express nearby genes, the tandem integrations of insertion vector and co-integration of mitochondrial DNA could impede the utilization of this promoter to activate genes near the insertion site. Testing of the HSP70 promoter was, therefore, not pursued.

Another novel addition to this insertion mutant library is the inclusion of unique barcodes. These barcodes allow one to take a census of the selected mutants by tracking barcode frequencies after the selection. Barcode sequencing can be facilitated by converting individual *Sfi* I site-bordered barcodes to barcode oligomers (Figure 2), which allows for the generation of multiple barcode sequences per Sanger sequencing reaction, providing a cost-effective and rapid alternative compared to cloning and sequencing individual barcodes. Although the high-throughput sequencing or microarray is possible analytic means for our mutant library, the design of our barcodes provides a medium-throughput alternative which requires only easily accessible laboratory techniques. Moreover, in contrast to the necessity of sophisticated bioinformatics support for microarray and high-throughput sequencing, the sequences of several hundred barcodes obtained from our approach can be easily sorted and analyzed with basic spreadsheet software. We note that our oligomerization and sequencing strategy can also be used to monitor other nucleic acids in cells (e.g. RNAs, mitochondrial genomes). By amplifying a small region around a sequence difference between two different nucleic acids, our oligomerization and sequencing method can easily produce ~200 or more sequences, which should be sufficient for determining the relative proportions of the two forms with data that are much easier to process than data from a high-throughput sequencing experiment.

Random insertion mutant libraries may overcome some challenges in the study of essential genes. Mutants that lack essential genes are not present in haploid gene deletion mutant banks such as the budding and fission yeast ORF deletion collections. Among the *S. pombe* insertion mutants analyzed in this work, we found that in six mutants, the insertions were identified in or adjacent to essential genes (Additional file 2: Table S1), presumably generating truncated proteins or altering the expression of these genes. These results indicate that this insertion mutant library approach provides opportunities for functional analysis of essential genes.

The insertion events characterized in this library are consistent with those shown in previous studies, including insertions in both genes and intergenic regions, large deletions of insertion DNA and little or no deletions of surrounding chromosomal sequences (Additional file 5: Table S2, and [20,21,25]). We also discovered 16 mutants with mitochondrial DNA co-integrated with the insertion vector. The presence of mitochondrial DNA in the wild type *S. pombe* nuclear genome has recently been characterized with one wild type strain containing 12 mitochondrial DNA insertions in its nuclear chromosomes [48]. Mitochondrial DNA fragments were also found in all repaired plasmid-based double strand breaks in *S. pombe* cells in an independent study [26]. It is worth noting that capture of mitochondrial DNA in the nuclear genome has also been observed in hemiascomycetous yeasts, plant, insect, rodent and human cells and appears to be an active and ongoing process [49-55], indicating that *S. pombe* transformation provides a way to study this process.

One consequence of mitochondrial and tandem *ura4*^+^ DNA insertions is that they can impede the detection of the insertion sites by TAIL-PCR. We have tested three additional approaches for mapping insertion mutations: splinkerette PCR, inverse splinkerette PCR and lox66/71-dependent cloning. While these methods were not 100% efficient, we showed that they could complement each other in defining insertion sites. One advantage of TAIL-PCR is that it detects the junction of insertion vector and genomic sequences and provides the exact location of the integrated vector. In contrast to TAIL-PCR, inverse splinkerette PCR in our mutants directly determined the closest *EcoR* V sites to the 5’ end (λ buffer end) of insertion vector. Together with the length of the PCR products, only approximate regions of insertion could be obtained. While we did not follow up results from our inverse splinkerette PCR, one could determine the exact location of insertion by cloning the PCR product and sequencing the genomic regions with gene-specific primers. Depending on the complexity of insertion structure, splinkerette PCR could determine the insertion vector-chromosome junction or the closest restriction sites used in the assay to the insertion site. It is important to note that all mutants are tagged by the lox71 sequence, which allows the cloning of the genomic sequences flanking the insertion in *E. coli* in the event that insertion mutations could not be mapped by these three PCR methods.

In addition to non-homologous recombination-based integration, other methods for generating insertion mutations include transposon-mediated mutagenesis. At least three types of transposons have been analyzed in a genome-wide context in *S. pombe*. The *S. pombe* retrotransposon Tf1 has been shown to exhibit preference for targeting the promoters of RNA polymerase II transcribed genes [56]. The *piggyBac* (*PB*) transposon, originally isolated from cabbage looper moth, preferably targets TTAA sites in the genome. Although as much as 79% of transposition events of *piggyBac* (*PB*) analyzed in a haploid *S. pombe* strain was located in intergenic regions, it was assumed that this seemingly preference of *PB* transposon for intergenic sequences was a consequence of selective pressure on insertions in ORFs that cause reduced fitness [57]. High throughput sequencing performed in both studies indicated that the transposition events of both transposons broadly distribute among the three chromosomes. The *Hermes* transposon from housefly *Musca domestica* has strong preference for T at position 2 and for A at position 7 of the target sites and has been adapted for *S. pombe*. The limited number of insertion mutants analyzed in that study suggested that *Hermes* targets both intergenic regions and coding sequences with no apparent bias [58]. Insertions generated by non-homologous recombination in our work also have a broad distribution on the three chromosomes, similar to what was observed in Tf1 retrotransposon and *PB* transposon [56,57] and a previous report on non-homologous recombination in *S. pombe*[20]. Although 60% (16/26) of the partially characterized insertions resided in ORFs or non-coding RNA genes, the enrichment of this type of insertions may be due to pre-selection of the corresponding mutants by visible phenotypes. Thus, the three transposon approaches and our insertion vector approach can create a wide variety of mutations. Our approach has the advantage of adding unique barcodes to each insertion, which has not been applied to the transposon approaches.

The main difference between non-homologous integration and transposon transposition is the structures of insertion events. While transposons generally integrate at individual genomic locations as unmodified single copies with defined junctions between genomic and transposon DNA, tandem integration of the insertion vector DNA or co-insertion of non-nuclear DNA during non-homologous recombination-based integration make this junction more variable. The simple insertion events in transposon mutagenesis allow for high-throughput sequencing for mapping insertion sites, while the complex insertion structures generated by non-homologous recombination require insertion sites to be determined by low/medium-throughput approaches. Nonetheless, the high mutation variety, the presence of random barcodes, and the availability of multiple methods for mapping insertion mutations still make this insertion mutant library an attractive tool for genome-wide studies that can complement the existing *S. pombe* ORF deletion set.

## Conclusions

As an effort to further support genome-wide studies in *S. pombe*, we generated a barcode-tagged *S. pombe* insertion mutant library which is available as pools of mixed mutants for parallel analysis and in the form of 384-well mutant arrays for genetic screens on individual mutants. The four genetic screens conducted in this work indicate that the library has a wide variety of mutations and is suited for the selection for diverse phenotypes. The design of the barcode tags allows detection and quantification of the barcodes with readily available molecular biological techniques, and does not require prior knowledge of the barcodes and mutations in the mutant strains to conduct genetic screens. The barcode-tagging strategy described here can be easily adapted to other model systems.

## Methods

### Strains and media

The *E. coli* electrocompetent cell NEB 5-alpha (Cat# C2989K, NEB) was used for the construction of the bacterial barcode-tagged insertion DNA library. The auxotrophic fission yeast wild type strain KRP1 [59] (originally designated as CHP429 from C. Hoffman [60]) was used to construct the fission yeast insertion mutant library.

Unless otherwise specified, yeast extract + 225 mg/l of supplements (YES) contains 3% glucose, and Edinburgh minimal medium (EMM) contains 2% glucose [61]. EMM + FOA contains 1 g/l of 5-FOA, 2% glucose, 50.25 mg/l of uracil [62]. Minimal medium agar (MMA) has 1% glucose [63]. For EMM, EMM + FOA and MMA media, the yeast complete supplements (YC – uracil) (Additional file 7: Table S3) were also included to allow the growth of additional auxotrophic mutants generated in this work.

### Construction of the bacterial barcode-tagged insertion DNA library

#### *Construction of the insertion DNA vector*

The protective λ buffer DNA and lexA-HSP70 promoter was created by PCR using nine overlapping oligonucleotides, hsplam1-9, and the primer S.pombeLmbdBrcd. Oligonucleotides used in this study are listed in Additional file 8: Table S4. This procedure removed all ATG codons in the transcribed strand of the λ buffer sequence. The final product (ATG-less λ-lexA-HSP70) contains the ATG-less λ buffer sequence, a mutated human HSP70 promoter, a lexA site and a 3’ *Blp* I site.

The selective marker *ura4*^*+*^ (InvU4) was first generated by PCR with primers InvU4S and InvU4-AS and wild type KRP1 genomic DNA as the template. A double strand lox71 DNA fragment was synthesized by annealing oligonucleotides lox71-InvU4S1 and lox71-InvU4AS1 and subsequent conversion of the annealed DNA to a double-strand product by Picomaxx (Agilent Technologies) on a thermal cycler by incubating the DNA-enzyme mixture at 94°C for 20 s with a decrease of 0.5°C per cycle for 72 cycles until the temperature reached 58°C. The double-strand lox71 DNA was used as a mega primer, together with the primer lox71-InvU4AS1, to amplify the InvU4 DNA by initial denaturation for 3 min at 94°C, then cycling 32 times with 30 s of denaturation (94°C), 30 s of annealing (48°C for the first 16 cycles, 52°C for the last 16 cycles) and 1 min and 45 s of extension (72°C), and a final extension step of 7 min at 72°C to generate a final DNA product (lox71-InvU4) that contains a 5’ *Sfi* I site, a lox71 site and the selectable marker *ura4*^+^.

The λ buffer fragment (ATG-less λ-lexA-HSP70) and the extended *ura4*^*+*^ marker (lox71-InvU4) were individually cloned to pCR2.1-TOPO vector (Invitrogen) and sequenced. The correct ATG-lessλ-lexA-HSP70 fragment was then purified as a *Hind* III-*EcoR* V fragment and ligated to pCR2.1-TOPO-lox71-InvU4 cut with *Hind* III and *Spe* I, where the *Spe* I overhang was rendered blunt by treatment with the Klenow fragment of *E. coli* DNA polymerase I (NEB), to make the final insertion vector construct pCR2.1-ATG-less λ-lexA-HSP70-lox71-InvU4, referred to as “pInsertion-ura4”. The complete sequence of pInsertion-ura4 is available as Additional file 9 (pCR2.1-TOPO sequence excluded).

#### *Preparing pInsertion-ura4 vector with a blunt end and a 5’ GGG overhang*

A 0.9 kb fragment containing part of the *sck1*^+^ coding sequence was generated by PCR using primers Stuffer 5’ *Blp* I and Stuffer 3’ *Sfi* I and wild type KRP1 genomic DNA as the template (PCR condition: initial denaturation for 3 min at 94°C and cycling 30 times with 20 s of denaturation (94°C), 30 s of annealing (56°C for the first 10 cycles, 52°C for the last 20 cycles) and 2 min of extension (72°C) followed by a final extension step of 5 min at 72°C). After digesting the PCR product with *Blp* I and *Sfi* I, the *sck1* stuffer fragment was inserted at the corresponding sites on pInsertion-ura4. The resulting stuffer plasmid (pInsertion-ura4-sck1) was digested with *Blp* I, treated with Klenow polymerase and then digested with *Sfi* I. The double-digested vector (6 kb) was separated from the *sck1* stuffer (0.9 kb) and partially-digested vector (6.9 kb) on a 0.7% agarose gel and purified.

#### *Insertion of the barcodes and bacterial library preparation*

The double strand barcode inserts were generated from two oligonucleotides, Barcode_3-07B and Barcode_P_3-07. Both oligonucleotides (20 μM) were separately heated at 70°C for 5 min, chilled quickly on ice and phosphorylated by T4 polynucleotide kinase (NEB) at 37°C for 1 h. The two phospho oligonucleotides were then annealed together by slow cooling on a thermal cycler using the following program: 95°C for 1 min and 30 s (−1°C/cycle, 15 cycles), 80°C for 2 min (−0.5°C/cycle, 70 cycles), 45°C × 1 min and 30 s (−0.5°C/cycle, 66 cycles). The annealed oligonucleotides were converted to double strand DNA by the Klenow fragment of *E. coli* DNA polymerase I (3’-5’ exo^-^) (NEB) at 37°C for 1 h, followed by 75°C incubation for 20 min to inactivate the enzyme. The resulting double strand barcodes had 5’ blunt ends and 3’ CCC overhangs that allowed their ligation to pInsertion-ura4 prepared above.

The pInsertion-ura4 vector and barcode DNA insert were ligated together at molar ratios of 1:1 (800 ng: 2.5 ng) or 1:3 (800 ng : 7.5 ng) by T4 DNA ligase (NEB) at 16°C for 16 h. Ligated DNA (20 ng or 40 ng) was transformed to 25 μl of *E. coli* electrocompetent cells NEB 5-alpha in a 1 mm electroporation cuvette on BioRad Gene Pulser II using the setting 1.7 kV, 200 Ω and 25 μF. After electroporation, 975 μl of SOC was added to the transformed cells, followed by incubation at 37°C with 250 rpm shaking for 1 h. To determine the titer of transformation, 3 μl of the culture was plated on LB + ampicillin (100 mg/l) plates in duplicate. For the remaining cells, aliquots of 100 μl were spread on one plate for a total of 10 plates and grown at 37°C for overnight. Cells grown on these plates were scraped off and grown in 100 ml of LB + ampicillin (100 mg/l) medium at 37°C for 4 h for plasmid DNA and freezer stock preparation.

#### Construction of the fission yeast barcode-tagged insertion mutant library

The linear insertion vector DNA was obtained by digesting the pInsertion-ura4-barcode library DNA with *BamH* I and gel purifying the 2.1 kb fragment using QIAGEN Gel Extraction kit, followed by extraction with phenol/chloroform/isoamyl alcohol (25:24:1; volume: volume: volume) and chloroform/isoamyl alcohol (24:1). For each transformation, 1 μg of purified linear barcoded insertion DNA was used to transform 50 μl of frozen KRP1 *S. pombe* competent cells (prepared as in [64]). Transformed cells were incubated at 30°C in all procedures described below.

Transformed cells were plated on MMA + YC − uracil + 5-FOA (0.1 g/l) (MMA + low FOA), where “YC − uracil” is the complete yeast supplements without uracil to allow recovery of auxotrophic mutants (Additional file 7: Table S3), and grown for at least 5 days. Colonies on MMA + low FOA plates were picked to grid on EMM + YC − uracil plates, grown for 3 days and replica plated onto YES plates. After growth on YES plates for 2 days, cells were replica plated to EMM + YC − uracil and EMM + YC + 5-FOA (1 g/l), in which the concentration of uracil is 50.25 mg/l (EMM + FOA). Cells that grew on EMM + YC − uracil but not on EMM + FOA plates were inoculated in 96-well plates with 200 μl of YES medium per well. As a second test for stable integration, selected transformants from each four 96-well plates were used to assemble 384-colony arrays on EMM + YC − uracil and EMM + FOA Omni plates (Nalge Nunc International) using a 96-floating-pin replicator and a colony copier (VP409 and VP381, V&P Scientific, Inc). Unstable transformants (i.e. cells that grew on EMM + FOA Omni plates) revealed in this step were removed from EMM + YC − uracil Omni plates.

Stable integrants that passed the second 5-FOA test were transferred to 40 μl of YES + 15% glycerol or EMM + YC − uracil + 15% glycerol medium in 384-well plates using a 384-floating-pin replicator (VP386, V&P Scientific, Inc) and incubated at 30°C for two days before being stored at −80°C. Cells left on each five EMM + YC − uracil Omni plates were scraped off and grown in a 250-ml flask with 50 ml of EMM + YC − uracil medium for 4 h at 30°C before aliquots were frozen in the presence of 15% glycerol as mixed library pools.

#### Determination of the size of *S. pombe* barcode-tagged insertion mutant library and bacterial barcode library

We chose to generate 10,000 *S. pombe* insertion mutants as a balance between the size of the yeast mutant library and the probability of obtaining a mutant in every protein coding gene, which was calculated by the sampling equation P = 1 − (1-f)^N^ where P is the probability of finding any genes in the genome, f is the fraction of a gene in the genome (gene size/genome size) and N is the number of insertion mutants generated [65]. Assuming that an insertion in the 5’, 3’ regions of a gene, introns and exons can produce a mutant phenotype, and the average size of a *S. pombe* gene is 2 kb in a genome of ~14,000 kb, the probability of finding at least a mutation in each individual gene in 10,000 random mutants P is 1 − (1 − 2/14,000)^10,000^ = 0.76 or 76%.

To increase the probability of tagging individual *S. pombe* insertion mutants with unique, non-redundant barcodes, only 250 to 1,500 *S. pombe* mutants were generated from each bacterial barcode sub-library, where the average number of barcode clones is ~1.86 × 10^5^. Thus, the number of *S. pombe* mutants generated from each sub-library corresponds to less than 1% of the available barcodes, and provides a ≥ 95% chance that all of the barcodes are unique. A total of 18 bacterial sub-libraries were used to generate and tag 10,000 *S. pombe* insertion mutants.

#### Barcode oligomerization

Barcode DNA with flanking insertion vector sequences (~ 760 bp) was amplified by PCR using genomic DNA prepared from fission yeast mutant cells in the library as the template, primers hsplam6 and BarcodePCR (888r), and the following program: initial denaturation for 3 min at 94°C, then cycling 30 times with 40 s of denaturation (94°C), 1 min of annealing (60°C for the first 10 cycles, 62°C for the last 20 cycles) and 1 min of extension (72°C), and a final extension step of 7 min at 72°C. After digestion of the PCR product with *Sfi* I enzyme, barcode DNA (66 bp) was separated from the two flanking DNA fragments (~200 and ~500 bp) on a 2% low melting agarose gel. The gel slice (~ 0.3 × 2 cm) containing the barcode DNA was melted at 65°C with 100 μl of 1X TE and 70 μl of 3 M sodium acetate, pH 5.2, and then extracted with 0.6 ml of TE-saturated phenol. The aqueous phase was re-extracted with 0.6 ml of phenol/chloroform/isoamyl alcohol, followed by extraction with 0.6 ml of chloroform/isoamyl alcohol. The final aqueous phase solution (~ 0.5 ml) was precipitated with 50 μl of 3 M sodium acetate, pH 5.2, 1.1 ml of 100% ethanol at −80°C overnight and the precipitated DNA was washed with 1 ml of 70% ethanol. The resulting barcode DNA was dissolved in 30 μl of 10 mM Tris–HCl, pH 8.0. Barcode DNA (~1 μg) was oligomerized by T4 DNA ligase (used 600 units at the beginning of the reaction and adding another 400 units after 8 h) with 15% polyethylene glycol (PEG) 3350 in a 20-μl reaction at 16°C for 16 h. The oligomerized barcode DNA was purified by QIAGEN PCR Purification kit to remove PEG and then resolved on a 2% low-melting agarose gel. Barcode oligomers with the size between 0.3 and 1 kb were purified using the method described above. The purified long barcode oligomers were ligated to *Sfi* I-digested and alkaline phosphatase (CIP)-treated pInsertion-ura4 vector and transformed to *E. coli*.

Bacterial transformants with large barcode inserts were first screened by extracting the total bacterial DNA from cells with phenol/chloroform/isoamyl alcohol and 1X DNA loading dye, and examining the aqueous phase, which contained bacterial genomic DNA and barcode-containing plasmids, by agarose gel electrophoresis to compare the electrophoretic mobility of barcode insert-containing plasmids (slow migrating) with the control plasmid without insert (pInsertion-ura4, fast migrating) on a 0.7% agarose gel. Plasmid DNA was purified from cells with large barcode inserts, verified by digestion with *BamH* I, and sequenced with primer TAIL-LB LOX71 to determine the barcode sequences.

#### Genetic screens to assess mutation diversity

Cells were first grown in 40 μl of YES medium in 384-well plates for two days. For the temperature sensitivity test, cells were transferred to two YES Omni plates, and one plate was incubated at 30°C and the other at 36°C. Temperature-sensitive mutants were scored as those that grew normally at 30°C but not (or slowly) at 36°C after incubation for 4 days. To identify mutants with slow or no growth on minimal medium, cells were transferred to EMM + adenine, histidine, leucine and EMM + YC − uracil Omni plates. Cells with auxotrophic mutations grew on EMM + YC − uracil but not EMM + adenine, histidine, leucine plates. For the identification of adenine biosynthesis mutations, cells were transferred to YES with low adenine Omni plates to look for mutants with altered colony color. To isolate EtBr-resistant mutations, cells grown in YES liquid medium were first inoculated to 40 μl of YES + 2% potassium acetate + 12.5 mg/l of ethidium bromide (YES + EtBr) medium [12] in 384-well plates for 2 days before transfer to YES + EtBr Omni plates. EtBr-resistant mutants were scored as the ability to grow on the YES + EtBr Omni plates. For the above three assays, cells were grown at 30°C for 5 days before scoring the phenotypes.

Mutants identified from the 384-colony array assays were individually verified by re-growing these mutants on the respective selective media or temperature as patches on regular Petri dish plates.

#### Generation of pLox66 plasmid and integration of pLox66 to *S. pombe* strains bearing lox71 sequence

The vector backbone was constructed by cutting pRS400 with *Pac* I and *Sac* II, rendering the ends blunt with Klenow enzyme and circularizing the vector by T4 DNA ligase. The double-strand lox66 DNA was generated by mixing and annealing oligonucleotides lox66_S and lox66_AS (25 μM each) on a thermal cycler by first denaturing at 95°C for three minutes and slow cooling from 95°C to 20°C (−0.5°C per cycle for 150 cycles with each cycle/temperature lasting for one minute). The resulting double strand DNA was ligated to the *Aat* II and *Bsa* I sites of pRS400ΔPacI/SacII (which deleted the ampicillin resistance gene) to generate the plasmid pLox66 (Additional file 10).

To integrate pLox66 into *S. pombe* cells bearing a chromosomal lox71 site, insertion mutant cells were first preloaded with Cre recombinase by transforming cells with pREP81-Cre [66] and growing transformed cells in EMM medium with 225 mg/l of adenine and histidine for 48 h. Cre recombinase-expressing cells were then transformed with pLox66 and maintained on solid EMM medium at 30°C for 24 h, followed by replica plating these cells to YES + G418 (200 mg/l) to select for cells with stably integrated pLox66.

#### Identification of insertion sites by thermal asymmetric interlaced (TAIL)-PCR

TAIL-PCR was conducted as described in Singer and Burke [41]. Briefly, three rounds of PCR, using alternate annealing temperatures, degenerate primers (TAIL AD1-6) and one of the three nested specific primers, TAIL LB2, TAIL lox71 and hsplam3 (or InversePCR1, InversePCR 3 and InversePCR 2), in each round, yielded one or a few bands in the tertiary PCR (Additional file 3: Figure S2A). The primary PCR used the genomic DNA of *S. pombe* insertion mutants as the template. One μl of 50-fold diluted products from the primary and the secondary PCR was used as the template in the secondary and the tertiary PCR, respectively. TAIL-PCR products were treated with exonuclease I and shrimp alkaline phosphatase (Exo-SAP, USB) or purified by QIAGEN Gel Extraction Kit prior to sequencing. The products from the secondary and the tertiary PCR were sequenced with primers hsplam5 and hsplam7 (or InversePCR 3 and InversePCR 2), respectively.

#### Identification of insertion sites by splinkerette PCR

Splinkerette PCR was carried out as described in [42,44]. Briefly, a double-stranded splinkerette adaptor with a hairpin-forming sequence and a *Spe* I/*Xba* I overhang was made by annealing oligos SPLK_A and SPLK_B_SpeI/XbaI on a thermal cycler by first denaturing at 95°C for three minutes and slow cooling from 95°C to 20°C (−0.5°C per cycle for 150 cycles with each cycle/temperature lasting for one minute). The splinkerette adaptor was ligated to genomic DNA digested with *Spe* I and *Xba* I, and the ligation product was used as the template in the first PCR reaction with primers SPLKFwd_1 and hsplam3. One μl of the 50-fold diluted first PCR product was used as the template in the second PCR with primers SPLKFwd_2 and hsplam5. The product generated in the second PCR reaction was sequenced by SPLKFwd_2 or hsplam5.

#### Identification of insertion sites by inverse splinkerette PCR

Genomic DNA (10 μg) was digested with 80 units of *EcoR* V-HF (NEB) at 37°C for 16 h, followed by 65°C denaturation for 15 min. A total of 1.5 μg of digested DNA was used in a 500-μl ligation (final DNA concentration = 3 ng/μl) with 2000 units of T4 DNA ligase at 16°C for 16 h. Ligated DNA was subsequently digested with 60 units of *Sfi* I restriction endonuclease at 50°C for five hours. All of the *Sfi* I-digested DNA was used in a 30-μl ligation reaction that contained 0.33 μM of double-strand *Sfi* I splinkerette and 800 units of T4 DNA ligase. The double-strand *Sfi* I splinkerette DNA was generated as described in the splinkerette PCR by annealing oligonucleotides Sfi I SPLK_A_GGG and Sfi I SPLK_B. After incubation at 16°C for 16 h, the ligation mixture was purified by phenol/chloroform extraction and ethanol precipitation as described above and resuspended in 10 μl of 10 mM Tris, pH 8.0.

To amplify genomic DNA flanked by *Sfi* I splinkerette and the *ura4*^+^ selectable marker, two rounds of PCR were carried out. In the first PCR, one μl of purified ligation product was used as the template with InvU4_1366F and SPLKFwd_1 primers. In the second PCR, one μl of 50-fold diluted first PCR product was used as the template with Ura4_EcoR V and SPLKFwd_2 primers. Products from the second PCR reaction were purified by QIAGEN Gel Purification Kit and sequenced by the primer Ura4_EcoR V.

## Competing interests

The authors declare that they have no competing interests.

## Authors’ contributions

B-RC and KWR designed the research project. B-RC, DCH and PJC carried out experiments. B-RC and KWR analyzed the data and wrote the manuscript. All authors have read and approved the final manuscript.

## Supplementary Material

Additional file 1 **Figure S1.** A strategy to enrich for cells that have few copies of the *ura4*^+^ gene. Based on a hypothetical metabolic outcome of altered Ura4 protein levels and low concentrations of 5-FOA on cell survival, cells bearing few or many copies of *ura4*^+^ genes are expected to exhibit different sensitivities to 5-FOA. (A) In cells with multiple copies of the *ura4*^+^ gene, increased levels of Ura4 allow efficient conversion of low dose of 5-FOA to toxic 5-fluorouracil. These cells die in the medium supplied with a low concentration of 5-FOA (i.e. 0.1 g/l, data not shown). (B) In cells bearing a small number of copies of the *ura4*^+^ gene, endogenous orotidine-5-phosophate (orotidine-5P) may outcompete 5-FOA supplied in low concentrations as the preferred substrate of the limited amount of Ura4, prevent Ura4 from metabolizing 5-FOA to 5-fluorouracil and allow such cells to grow in medium with low 5-FOA. Click here for file

Additional file 2 **Table S1.** Tetrad analysis and insertion site mapping of *S. pombe* barcoded insertion mutants. Click here for file

Additional file 3 **Figure S2.** Characterization of insertion events by thermal asymmetric interlaced (TAIL)-PCR. (A) In mutants with a single or very few copies of insertion vectors, DNA fragments composed of partial insertion vector sequences and adjacent genomic DNA sequences could be amplified using vector-specific primers and a mixture of arbitrary degenerate (AD) primers. The nested vector-specific primers in each round of PCR reactions allowed for amplification of the final PCR products with increasing specificity. (B) In the event of tandem integration of multiple copies of the insertion vector, TAIL-PCR often resulted in amplification within insertion vectors due to additional binding sites for AD primers in the additional copies of insertion vectors. When vectors were integrated in head-to-head orientation, vector-specific primers could bind both strands of the repetitive insertion vector DNA and only amplify the vector sequences. (C) Mitochondrial DNA was found co-integrated with the insertion vector into the genome in some mutants. In such mutants, TAIL-PCR could only amplify DNA sequences corresponding to the insertion vector and mitochondrial DNA, which might result from additional binding site for AD primers in mitochondrial DNA. Click here for file

Additional file 4 **Figure S3.** Characterization of insertion events by splinkerette PCR. In splinkerette PCR, genomic DNA is first digested with restriction enzymes that do not cut or cut very few times in the insertion vector (e.g. *Xba* I and *Spe* I, or *Bcl* I and *Bgl* II). Double strand splinkerette adaptor DNA can then be ligated to the digested genomic DNA with compatible overhangs. The resulting products, genomic fragments flanked by splinkerette and insertion vector DNA, can be amplified by PCR using primers on splinkerette (solid arrows) and the insertion vector (dashed arrows). Click here for file

Additional file 5 **Table S2.** Insertion events with both insertion-genomic junctions characterized. Click here for file

Additional file 6 Potential modes of integration of non-homologous DNA in *S. pombe* genome. After being transfected into *S. pombe* cells, non-homologous DNA could be integrated into the genome as a single copy. In some cases, the ends of non-homologous DNA may be first deleted by nucleolytic activities in cells and then ligated to other linear DNA fragments in head-to-tail, heat-to-head or tail-to-tail orientations before being integrated in the genome. Click here for file

Additional file 7 **Table S3.** The amino acid and nucleobase supplements in the minimum medium + YC – uracil. Click here for file

Additional file 8 **Table S4.** Oligonucleotides used in this study. Click here for file

Additional file 9 **pInsertion-ura4.** Annotated sequence of pInsertion-ura4. Click here for file

Additional file 10 **pLox66.** Annotated sequence of pLox66. Click here for file

## References

[B1] Schena M Shalon D Davis RW Brown PO Quantitative monitoring of gene expression patterns with a complementary DNA microarray Science 1995 270 5235 467 470 10.1126/science.270.5235.467 7569999

[B2] Giaever G Chu AM Ni L Connelly C Riles L Veronneau S Dow S Lucau-Danila A Anderson K Andre B Functional profiling of the Saccharomyces cerevisiae genome Nature 2002 418 6896 387 391 10.1038/nature00935 12140549

[B3] Hensel M Shea JE Gleeson C Jones MD Dalton E Holden DW Simultaneous identification of bacterial virulence genes by negative selection Science 1995 269 5222 400 403 10.1126/science.7618105 7618105

[B4] Shoemaker DD Lashkari DA Morris D Mittmann M Davis RW Quantitative phenotypic analysis of yeast deletion mutants using a highly parallel molecular bar-coding strategy Nat Genet 1996 14 4 450 456 10.1038/ng1296-450 8944025

[B5] Singhi AD Kondratov RV Neznanov N Chernov MV Gudkov AV Selection-subtraction approach (SSA): a universal genetic screening technique that enables negative selection Proc Natl Acad Sci U S A 2004 101 25 9327 9332 10.1073/pnas.0403080101 15187233 PMC438976

[B6] Ngo VN Davis RE Lamy L Yu X Zhao H Lenz G Lam LT Dave S Yang L Powell J A loss-of-function RNA interference screen for molecular targets in cancer Nature 2006 441 7089 106 110 10.1038/nature04687 16572121

[B7] Winzeler EA Shoemaker DD Astromoff A Liang H Anderson K Andre B Bangham R Benito R Boeke JD Bussey H Functional characterization of the S. cerevisiae genome by gene deletion and parallel analysis Science 1999 285 5429 901 906 10.1126/science.285.5429.901 10436161

[B8] Fabrizio P Hoon S Shamalnasab M Galbani A Wei M Giaever G Nislow C Longo VD Genome-wide screen in Saccharomyces cerevisiae identifies vacuolar protein sorting, autophagy, biosynthetic, and tRNA methylation genes involved in life span regulation PLoS Genet 2010 6 7 e1001024 10.1371/journal.pgen.1001024 20657825 PMC2904796

[B9] Matecic M Smith DL Pan X Maqani N Bekiranov S Boeke JD Smith JS A microarray-based genetic screen for yeast chronological aging factors PLoS Genet 2010 6 4 e1000921 10.1371/journal.pgen.1000921 20421943 PMC2858703

[B10] Wood V Gwilliam R Rajandream MA Lyne M Lyne R Stewart A Sgouros J Peat N Hayles J Baker S The genome sequence of Schizosaccharomyces pombe Nature 2002 415 6874 871 880 10.1038/nature724 11859360

[B11] Forsburg SL Rhind N Basic methods for fission yeast Yeast 2006 23 3 173 183 10.1002/yea.1347 16498704 PMC5074380

[B12] Haffter P Fox TD Nuclear mutations in the petite-negative yeast Schizosaccharomyces pombe allow growth of cells lacking mitochondrial DNA Genetics 1992 131 2 255 260 1644270 10.1093/genetics/131.2.255 PMC1205001

[B13] Kaufer NF Potashkin J Analysis of the splicing machinery in fission yeast: a comparison with budding yeast and mammals Nucleic Acids Res 2000 28 16 3003 3010 10.1093/nar/28.16.3003 10931913 PMC108416

[B14] Kaufer NF Simanis V Nurse P Fission yeast Schizosaccharomyces pombe correctly excises a mammalian RNA transcript intervening sequence Nature 1985 318 6041 78 80 10.1038/318078a0 2997624

[B15] Shuster EO Guthrie C Human U2 snRNA can function in pre-mRNA splicing in yeast Nature 1990 345 6272 270 273 10.1038/345270a0 2185425

[B16] Volpe TA Kidner C Hall IM Teng G Grewal SI Martienssen RA Regulation of heterochromatic silencing and histone H3 lysine-9 methylation by RNAi Science 2002 297 5588 1833 1837 10.1126/science.1074973 12193640

[B17] Kim DU Hayles J Kim D Wood V Park HO Won M Yoo HS Duhig T Nam M Palmer G Analysis of a genome-wide set of gene deletions in the fission yeast Schizosaccharomyces pombe Nat Biotechnol 2010 28 6 617 623 10.1038/nbt.1628 20473289 PMC3962850

[B18] Smith JS Caputo E Boeke JD A genetic screen for ribosomal DNA silencing defects identifies multiple DNA replication and chromatin-modulating factors Mol Cell Biol 1999 19 4 3184 3197 10082585 10.1128/mcb.19.4.3184 PMC84112

[B19] Curran SP Ruvkun G Lifespan regulation by evolutionarily conserved genes essential for viability PLoS Genet 2007 3 4 e56 10.1371/journal.pgen.0030056 17411345 PMC1847696

[B20] Chua G Taricani L Stangle W Young PG Insertional mutagenesis based on illegitimate recombination in Schizosaccharomyces pombe Nucleic Acids Res 2000 28 11 E53 10.1093/nar/28.11.e53 10871352 PMC102638

[B21] Davidson MK Young NP Glick GG Wahls WP Meiotic chromosome segregation mutants identified by insertional mutagenesis of fission yeast Schizosaccharomyces pombe; tandem-repeat, single-site integrations Nucleic Acids Res 2004 32 14 4400 4410 10.1093/nar/gkh767 15316103 PMC514387

[B22] Schuldiner M Collins SR Thompson NJ Denic V Bhamidipati A Punna T Ihmels J Andrews B Boone C Greenblatt JF Exploration of the function and organization of the yeast early secretory pathway through an epistatic miniarray profile Cell 2005 123 3 507 519 10.1016/j.cell.2005.08.031 16269340

[B23] Muhlrad D Parker R Aberrant mRNAs with extended 3' UTRs are substrates for rapid degradation by mRNA surveillance RNA 1999 5 10 1299 1307 10.1017/S1355838299990829 10573121 PMC1369852

[B24] Grimm C Kohli J Observations on integrative transformation in Schizosaccharomyces pombe Mol Gen Genet 1988 215 1 87 93 10.1007/BF00331308 3241625

[B25] Tatebayashi K Kato J Ikeda H Structural analyses of DNA fragments integrated by illegitimate recombination in Schizosaccharomyces pombe Mol Gen Genet 1994 244 2 111 119 8052229 10.1007/BF00283511

[B26] Decottignies A Capture of extranuclear DNA at fission yeast double-strand breaks Genetics 2005 171 4 1535 1548 10.1534/genetics.105.046144 16143617 PMC1456082

[B27] Boeke JD LaCroute F Fink GR A positive selection for mutants lacking orotidine-5'-phosphate decarboxylase activity in yeast: 5-fluoro-orotic acid resistance Mol Gen Genet 1984 197 2 345 346 10.1007/BF00330984 6394957

[B28] Grimm C Kohli J Murray J Maundrell K Genetic engineering of Schizosaccharomyces pombe: a system for gene disruption and replacement using the ura4 gene as a selectable marker Mol Gen Genet 1988 215 1 81 86 10.1007/BF00331307 3241624

[B29] Prentice HL Kingston RE Mammalian promoter element function in the fission yeast Schizosaccharomyces pombe Nucleic Acids Res 1992 20 13 3383 3390 10.1093/nar/20.13.3383 1321414 PMC312493

[B30] Walker GC Mutagenesis and inducible responses to deoxyribonucleic acid damage in Escherichia coli Microbiol Rev 1984 48 1 60 93 6371470 10.1128/mr.48.1.60-93.1984 PMC373003

[B31] Araki K Araki M Yamamura K Targeted integration of DNA using mutant lox sites in embryonic stem cells Nucleic Acids Res 1997 25 4 868 872 10.1093/nar/25.4.868 9016639 PMC146486

[B32] Vojtek AB Hollenberg SM Cooper JA Mammalian Ras interacts directly with the serine/threonine kinase Raf Cell 1993 74 1 205 214 10.1016/0092-8674(93)90307-C 8334704

[B33] Schizosaccharomyces pombe GeneDB http://old.genedb.org/amigo-cgi/browse.cgi?speciesdb=GeneDB_Spombe

[B34] Dujon B Strathern JN, Jones EW, Broach JR Mitochondrial genetics and functions The Molecular Biology of the Yeast Saccharomyces 1981 Cold Spring Harbor Laboratory Press, Cold Spring Harbor, NY 505 635

[B35] Kim G Sikder H Singh KK A colony color method identifies the vulnerability of mitochondria to oxidative damage Mutagenesis 2002 17 5 375 381 10.1093/mutage/17.5.375 12202624

[B36] Kyoto Encyclopedia of Genes and Genomes http://www.genome.jp/kegg/

[B37] King MP Attardi G Human cells lacking mtDNA: repopulation with exogenous mitochondria by complementation Science 1989 246 4929 500 503 10.1126/science.2814477 2814477

[B38] Saccharomyces Genome Deletion Project http://www-sequence.stanford.edu/group/yeast_deletion_project/project_desc.html

[B39] Stansfield I Stark MJR Yeast Gene Analysis 2007 Elsevier Academic Press,

[B40] Liu YG Whittier RF Thermal asymmetric interlaced PCR: automatable amplification and sequencing of insert end fragments from P1 and YAC clones for chromosome walking Genomics 1995 25 3 674 681 10.1016/0888-7543(95)80010-J 7759102

[B41] Singer T Burke E High-throughput TAIL-PCR as a tool to identify DNA flanking insertions Methods Mol Biol 2003 236 241 272 14501069 10.1385/1-59259-413-1:241

[B42] Horn C Hansen J Schnutgen F Seisenberger C Floss T Irgang M De-Zolt S Wurst W von Melchner H Noppinger PR Splinkerette PCR for more efficient characterization of gene trap events Nat Genet 2007 39 8 933 934 10.1038/ng0807-933 17660805

[B43] Uren AG Mikkers H Kool J van der Weyden L Lund AH Wilson CH Rance R Jonkers J van Lohuizen M Berns A A high-throughput splinkerette-PCR method for the isolation and sequencing of retroviral insertion sites Nat Protoc 2009 4 5 789 798 10.1038/nprot.2009.64 19528954 PMC3627465

[B44] Devon RS Porteous DJ Brookes AJ Splinkerettes–improved vectorettes for greater efficiency in PCR walking Nucleic Acids Res 1995 23 9 1644 1645 10.1093/nar/23.9.1644 7784225 PMC306912

[B45] Roguev A Wiren M Weissman JS Krogan NJ High-throughput genetic interaction mapping in the fission yeast Schizosaccharomyces pombe Nature methods 2007 4 10 861 866 10.1038/nmeth1098 17893680

[B46] Tong AH Evangelista M Parsons AB Xu H Bader GD Page N Robinson M Raghibizadeh S Hogue CW Bussey H Systematic genetic analysis with ordered arrays of yeast deletion mutants Science 2001 294 5550 2364 2368 10.1126/science.1065810 11743205

[B47] Gottschling DE Aparicio OM Billington BL Zakian VA Position effect at S. cerevisiae telomeres: reversible repression of Pol II transcription. Cell 1990 63 4 751 762 10.1016/0092-8674(90)90141-z 2225075

[B48] Lenglez S Hermand D Decottignies A Genome-wide mapping of nuclear mitochondrial DNA sequences links DNA replication origins to chromosomal double-strand break formation in Schizosaccharomyces pombe Genome Res 2010 20 9 1250 1261 10.1101/gr.104513.109 20688779 PMC2928503

[B49] Behura SK Analysis of nuclear copies of mitochondrial sequences in honeybee (Apis mellifera) genome Molecular biology and evolution 2007 24 7 1492 1505 10.1093/molbev/msm068 17404397

[B50] Pamilo P Viljakainen L Vihavainen A Exceptionally high density of NUMTs in the honeybee genome Molecular biology and evolution 2007 24 6 1340 1346 10.1093/molbev/msm055 17383971

[B51] Cheng X Ivessa AS The migration of mitochondrial DNA fragments to the nucleus affects the chronological aging process of Saccharomyces cerevisiae Aging Cell 2010 9 5 919 923 10.1111/j.1474-9726.2010.00607.x 20626726 PMC3394387

[B52] Sacerdot C Casaregola S Lafontaine I Tekaia F Dujon B Ozier-Kalogeropoulos O Promiscuous DNA in the nuclear genomes of hemiascomycetous yeasts FEMS Yeast Res 2008 8 6 846 857 10.1111/j.1567-1364.2008.00409.x 18673395

[B53] Hazkani-Covo E Zeller RM Martin W Molecular poltergeists: mitochondrial DNA copies (numts) in sequenced nuclear genomes PLoS Genet 2010 6 2 e1000834 10.1371/journal.pgen.1000834 20168995 PMC2820518

[B54] Triant DA DeWoody JA Molecular analyses of mitochondrial pseudogenes within the nuclear genome of arvicoline rodents Genetica 2008 132 1 21 33 17333478 10.1007/s10709-007-9145-6

[B55] Noutsos C Richly E Leister D Generation and evolutionary fate of insertions of organelle DNA in the nuclear genomes of flowering plants Genome Res 2005 15 5 616 628 10.1101/gr.3788705 15867426 PMC1088290

[B56] Guo Y Levin HL High-throughput sequencing of retrotransposon integration provides a saturated profile of target activity in Schizosaccharomyces pombe Genome Res 2010 20 2 239 248 10.1101/gr.099648.109 20040583 PMC2813479

[B57] Li J Zhang JM Li X Suo F Zhang MJ Hou W Han J Du LL A piggyBac transposon-based mutagenesis system for the fission yeast Schizosaccharomyces pombe Nucleic Acids Res 2011 39 6 e40 10.1093/nar/gkq1358 21247877 PMC3064801

[B58] Evertts AG Plymire C Craig NL Levin HL The hermes transposon of Musca domestica is an efficient tool for the mutagenesis of Schizosaccharomyces pombe Genetics 2007 177 4 2519 2523 10.1534/genetics.107.081075 17947404 PMC2219505

[B59] Chen BR Runge KW A new Schizosaccharomyces pombe chronological lifespan assay reveals that caloric restriction promotes efficient cell cycle exit and extends longevity Exp Gerontol 2009 44 8 493 502 10.1016/j.exger.2009.04.004 19409973 PMC2795633

[B60] Apolinario E Nocero M Jin M Hoffman CS Cloning and manipulation of the Schizosaccharomyces pombe his7+ gene as a new selectable marker for molecular genetic studies Curr Genet 1993 24 6 491 495 10.1007/BF00351711 8299169 PMC4417482

[B61] Moreno S Klar A Nurse P Molecular Biology of the Fission Yeast Schizosacchromyces pombe MethEnzym 1991 194 795 823 10.1016/0076-6879(91)94059-l 2005825

[B62] PombeNet http://www-bcf.usc.edu/~forsburg/drugs.html#FOA

[B63] Hirashima K Iwaki T Takegawa K Giga-Hama Y Tohda H A simple and effective chromosome modification method for large-scale deletion of genome sequences and identification of essential genes in fission yeast Nucleic Acids Res 2006 34 2 e11 10.1093/nar/gnj011 16434698 PMC1351375

[B64] Suga M Hatakeyama T A rapid and simple procedure for high-efficiency lithium acetate transformation of cryopreserved Schizosaccharomyces pombe cells Yeast 2005 22 10 799 804 10.1002/yea.1247 16088874

[B65] Sambrook J Russell DW Molecular Cloning-A laboratory manual 2001 vol. 1, 3 Cold Spring Harbor Laboratory Press, Cold Spring Harbor, New York

[B66] Molnar M Kleckner N Examination of interchromosomal interactions in vegetatively growing diploid Schizosaccharomyces pombe cells by Cre/loxP site-specific recombination Genetics 2008 178 1 99 112 10.1534/genetics.107.082826 18202361 PMC2206114

