# Supplementary figures and images for: Generation and analysis of a barcode-tagged insertion mutant library in the fission yeast Schizosaccharomyces pombe
Source: BMC Genomics. 2012 May 3;13:161. doi: 10.1186/1471-2164-13-161 (PMC3418178; doi:10.1186/1471-2164-13-161)

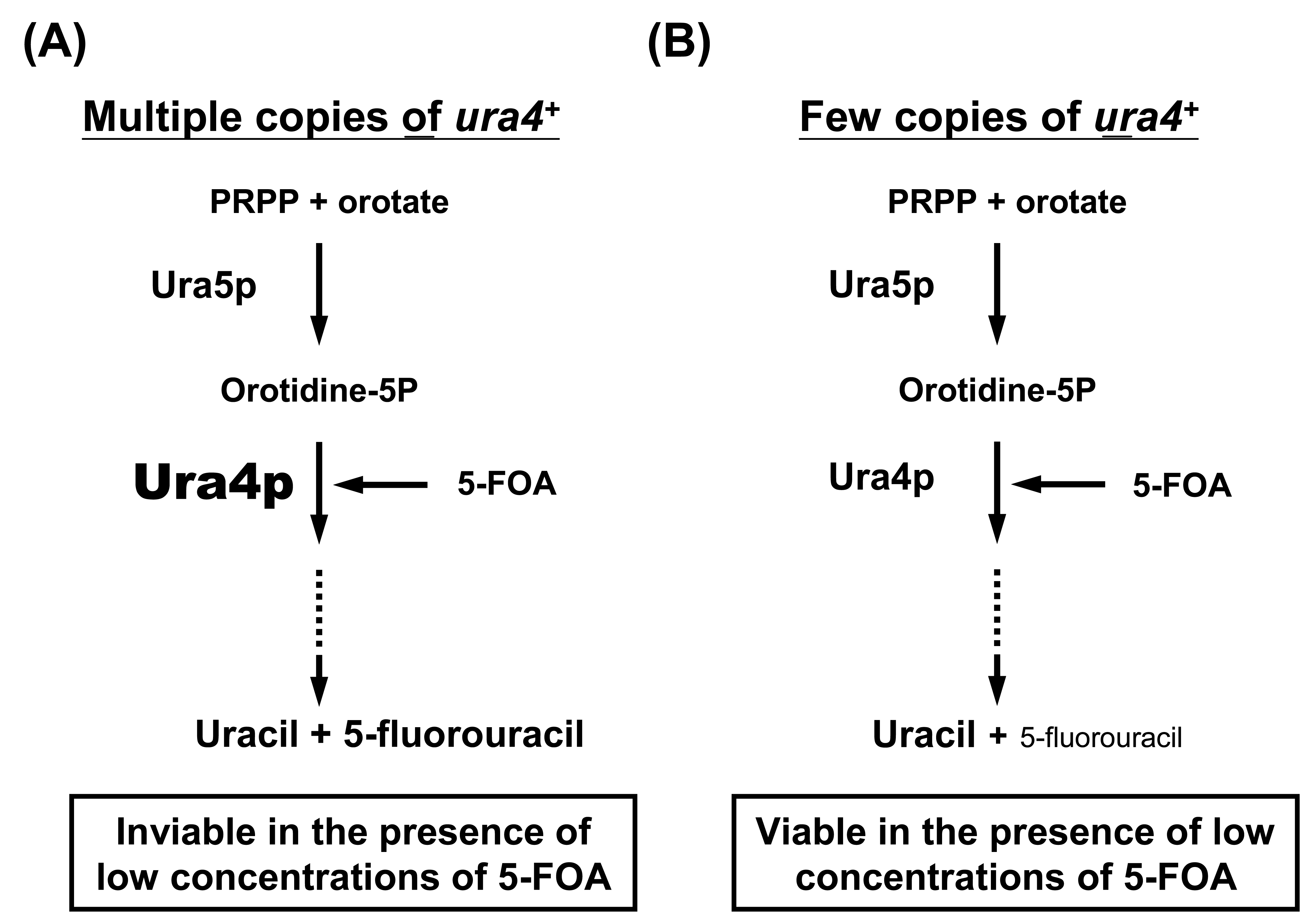

Supplement: Additional file 1 — Figure S1. A strategy to enrich for cells that have few copies of the ura4+ gene. Based on a hypothetical metabolic outcome of altered Ura4 protein levels and low concentrations of 5-FOA on cell survival, cells bearing few or many copies of ura4+ genes are expected to exhibit different sensitivities to 5-FOA. (A) In cells with multiple copies of the ura4+ gene, increased levels of Ura4 allow efficient conversion of low dose of 5-FOA to toxic 5-fluorouracil. These cells die in the medium supplied with a low concentration of 5-FOA (i.e. 0.1 g/l, data not shown). (B) In cells bearing a small number of copies of the ura4+ gene, endogenous orotidine-5-phosophate (orotidine-5P) may outcompete 5-FOA supplied in low concentrations as the preferred substrate of the limited amount of Ura4, prevent Ura4 from metabolizing 5-FOA to 5-fluorouracil and allow such cells to grow in medium with low 5-FOA. [file 1471-2164-13-161-S1.jpeg]

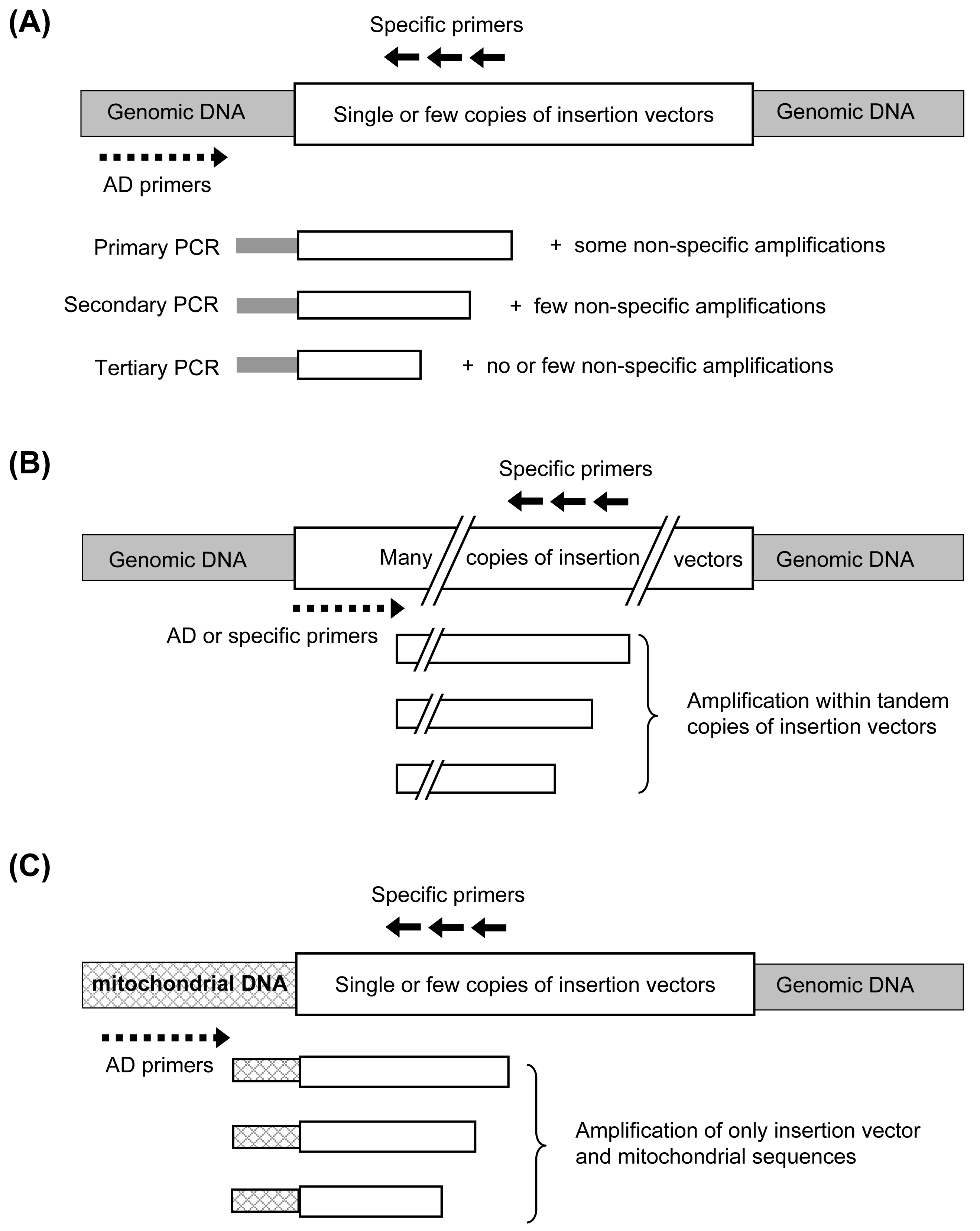

Supplement: Additional file 3 — Figure S2. Characterization of insertion events by thermal asymmetric interlaced (TAIL)-PCR. (A) In mutants with a single or very few copies of insertion vectors, DNA fragments composed of partial insertion vector sequences and adjacent genomic DNA sequences could be amplified using vector-specific primers and a mixture of arbitrary degenerate (AD) primers. The nested vector-specific primers in each round of PCR reactions allowed for amplification of the final PCR products with increasing specificity. (B) In the event of tandem integration of multiple copies of the insertion vector, TAIL-PCR often resulted in amplification within insertion vectors due to additional binding sites for AD primers in the additional copies of insertion vectors. When vectors were integrated in head-to-head orientation, vector-specific primers could bind both strands of the repetitive insertion vector DNA and only amplify the vector sequences. (C) Mitochondrial DNA was found co-integrated with the insertion vector into the genome in some mutants. In such mutants, TAIL-PCR could only amplify DNA sequences corresponding to the insertion vector and mitochondrial DNA, which might result from additional binding site for AD primers in mitochondrial DNA. [file 1471-2164-13-161-S3.jpeg]

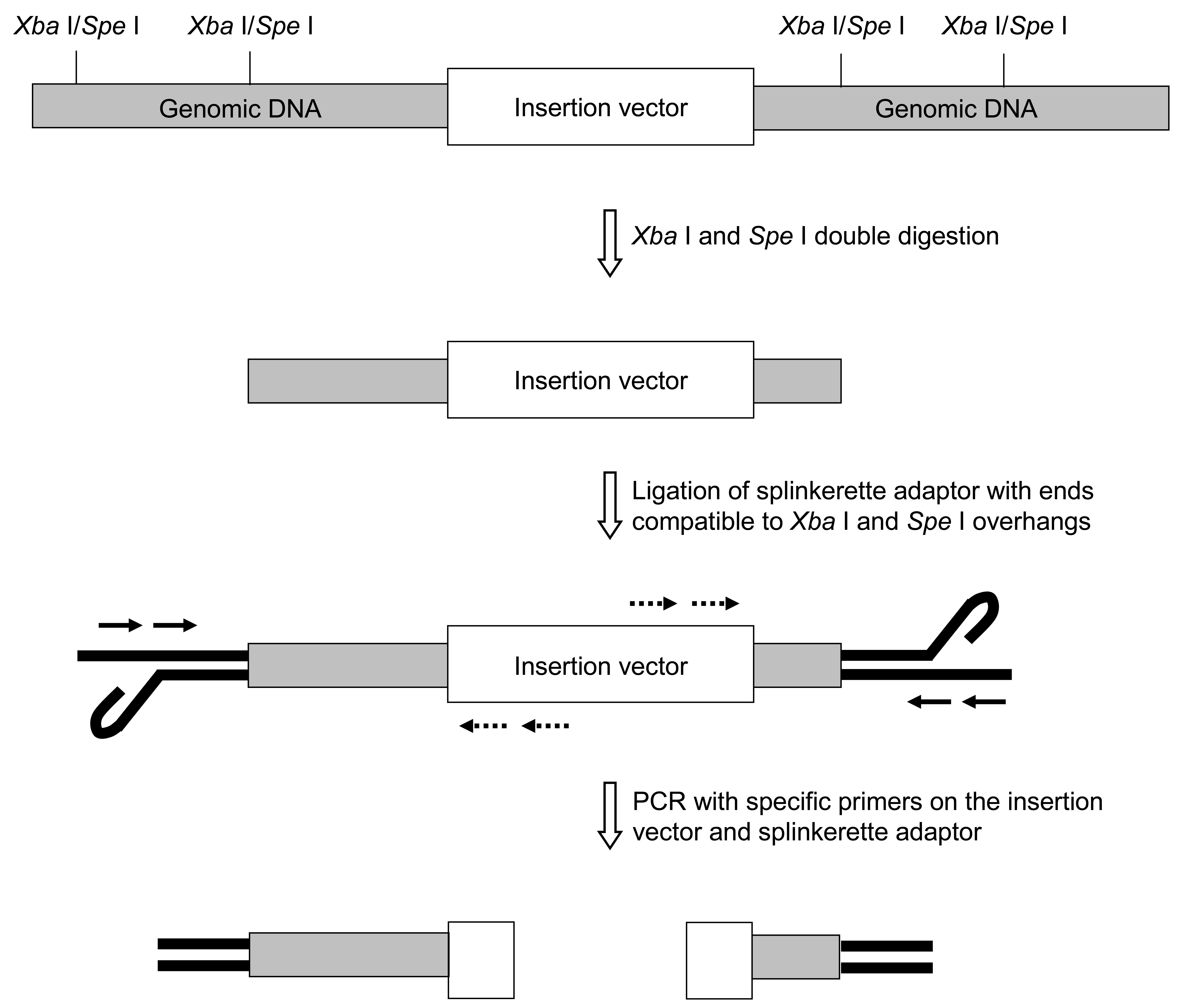

Supplement: Additional file 4 — Figure S3. Characterization of insertion events by splinkerette PCR. In splinkerette PCR, genomic DNA is first digested with restriction enzymes that do not cut or cut very few times in the insertion vector (e.g. Xba I and Spe I, or Bcl I and Bgl II). Double strand splinkerette adaptor DNA can then be ligated to the digested genomic DNA with compatible overhangs. The resulting products, genomic fragments flanked by splinkerette and insertion vector DNA, can be amplified by PCR using primers on splinkerette (solid arrows) and the insertion vector (dashed arrows). [file 1471-2164-13-161-S4.jpeg]

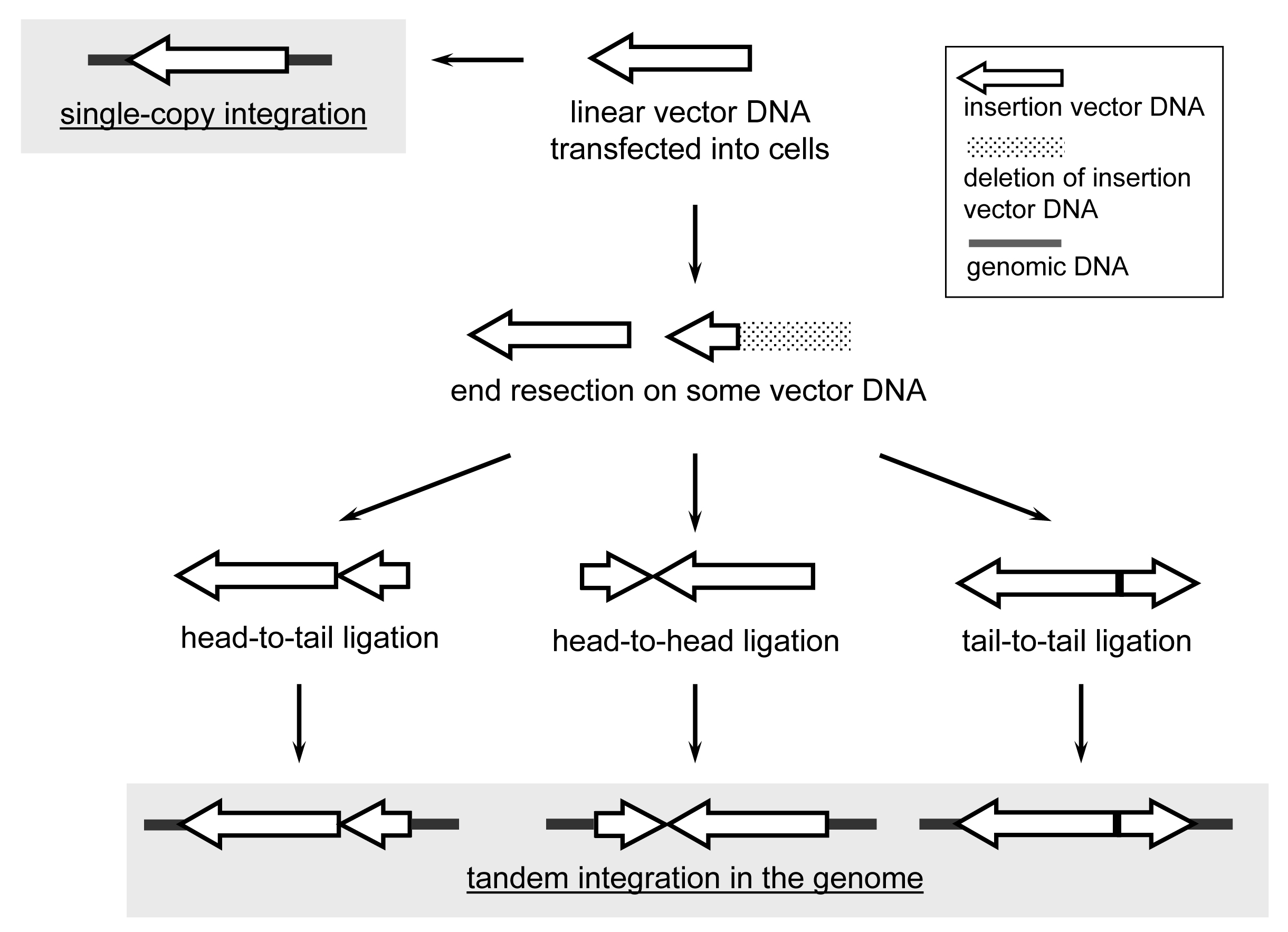

Supplement: Additional file 6 — Potential modes of integration of non-homologous DNA in S. pombe genome. After being transfected into S. pombe cells, non-homologous DNA could be integrated into the genome as a single copy. In some cases, the ends of non-homologous DNA may be first deleted by nucleolytic activities in cells and then ligated to other linear DNA fragments in head-to-tail, heat-to-head or tail-to-tail orientations before being integrated in the genome. [file 1471-2164-13-161-S6.jpeg]
