# Supplementary material for: Generation and analysis of a barcode-tagged insertion mutant library in the fission yeast Schizosaccharomyces pombe
Source: BMC Genomics. 2012 May 3;13:161. doi: 10.1186/1471-2164-13-161 (PMC3418178; doi:10.1186/1471-2164-13-161)
Supplement: Additional file 5 — Table S2. Insertion events with both insertion-genomic junctions characterized. [file 1471-2164-13-161-S5.pdf]

Table S2. Insertion events with both insertion-genomic junctions characterized

Mutant	Insertion site (Chromosome)	Deletion (bp) in : genome	5' of vector	3' of vector	Tandem insertion
13_C10	Chromosome 2; 1157467* -1157468	0	391 (1st copy)	1093 (last copy)	Yes (≥ 4 copies)
13_H5	Chromosome 1; 1548917-1548923*	5	11 (1st copy)	5 (last copy)	Yes (≥ 3 copies)
18_M24	Chromosome 1; 4748287*-4748290	2	10 (1st copy)<br>231 (2nd copy)<br>14 (3rd copy)	4 (1st copy)<br>1834 (2nd copy)<br>1847 (3rd copy)	Yes (3 copies)
1a7-4033	Chromosome 2; 1746832*-1746837	4	11 (1st copy)<br>10 (2nd copy)	5 (1st copy)<br>976 (2nd copy)	Yes (2 copies)
1a8-4032	Chromosome 1; 214745*-214746	0	36	17	No
1a8-6535	Chromosome 3; 1823350-1823351*	0	10	3	Yes[a]
10a1-36668	Chromosome 3; 1774653-1774654*	0	9 (1st copy)	6 (last copy)	Yes (≥ 2 copies)

The asterisks (*) indicate the ends of insertion sites mapped by TAIL-PCR. The other ends were determined by PCR with flanking primers of the mapped insertion sites.

a. PCRs with gene-specific primers flanking the insertion sites generated products larger the size of DNA with a single copy of the insertion vector.
