# Supplementary material for: Generation and analysis of a barcode-tagged insertion mutant library in the fission yeast Schizosaccharomyces pombe
Source: BMC Genomics. 2012 May 3;13:161. doi: 10.1186/1471-2164-13-161 (PMC3418178; doi:10.1186/1471-2164-13-161)
Supplement: Additional file 7 — Table S3. The amino acid and nucleobase supplements in the minimum medium + YC – uracil. [file 1471-2164-13-161-S7.pdf]

Table S3. The amino acid and nucleobase supplements in the minimum medium + YC - uracil

Supplement	mg/l	Supplement	mg/l	Supplement	mg/l	Supplement	mg/l
lysine	150	tryptophan	150	isoleucine	75	adenine	225
arginine	150	tyrosine	75	asparagine	75	leucine	225
threonine	150	methionine	75	proline	75	histidine	225
cysteine	150	valine	75	phenylalanine	75		
