# Supplementary material for: Generation and analysis of a barcode-tagged insertion mutant library in the fission yeast Schizosaccharomyces pombe
Source: BMC Genomics. 2012 May 3;13:161. doi: 10.1186/1471-2164-13-161 (PMC3418178; doi:10.1186/1471-2164-13-161)
Supplement: Additional file 8 — Table S4. Oligonucleotides used in this study. [file 1471-2164-13-161-S8.pdf]

Table S4. Oligonucleotides used in this study

Oligonucleotide name	Sequence
Construction of ATGless λ buffer-HSP70 sequence	
hsplam1	GCTGAGCGCTAGCTACTGTATGTACATACAGTACCTCATCGAGCTCGGTT
hsplam2	GGAGAGTCGACTTTTCCCTTCTGACTGCCTAACCGAGCTCGATGAGGTAC
hsplam3	AAGGGAAAAGTCGACTCTCCGTGACGACTTATAAAAGCCCAGGGGCAAG
hsplam4	CACCGTTAGCCGTTATCCGGACCGCTTGCCCCTGGGCTTTTATAAG
hsplam5	GGATAACGGCTAACGGTGTACGTCAGCCCGGAAAAGTGCATATCCAG
hsplam6	GTACCTAATATTTTCACGATGTTCTGCTGGATATGCACTTTTCCGGGCTG
hsplam7	CATCGTGAAAATATTAGGTACTGTAAAAGCGGTGCCAGTCGGCATAC
hsplam8	GCCGGGATGTGATCCACGGAGTATGCCGACTGGCACCGCTTTTAC
hsplam9	TCCGTGGATCACATCCCGGCAAGCTTGGCACGCCAGTCGGGT
S.pombeLmbdBrcd	TCCGGATCCGTTTCTGCGGGAAAG
Construction of lox71-InvU4	
lox71-InvU4S1	CCATGGCCTCCCTGGCCTACCGTTCGTATAGCATACATTATACGAAG
lox71-InvU4AS1	CACAAATGCATACATATAGCCAGTGGATAACTTCGTATAATGTATGCTATAC
InvU4S	CACTGGCTATATGTATGCATTTGTG
InvU4AS	TCCGGATCCCGAAACTTTTTGACATCTAATTTATTCTGTTCC
Generation of sck1 stuffer	
Stuffer 5' *Blp* I	TACAGCTCAGCTTCACAAAGAACAGG
Stuffer 3' *Sfi* I	TGAGGCCAGGGAGGCCTACGGACCAAATAAACTTGCCC
Generation of double strand barcode inserts	
Barcode_P_3-07	AGGCCCGGGCGAGTGT
Barcode_3-07B†	GCCTGGCCTCCCTGGCCANNNANANNNANANNNANANNNANANNNANANNNA NANNNACACTCGCCCGGGCCTCCC
TAIL PCR primers	
TAIL-LB2	CTCCATTAAGTAACAAATTCCTATTTAGAGAAAGAATGCTGAGTA
TAIL-LB LOX71	AGCCAGTGGATAACTTCGTATAATGTATGCTATACGAACGGTA
InversePCR 1	GGAAGGCATATCAGCAAAGACTTTCTCAGC
InversePCR 2	ACATGCTCCTACAACATTACCACAATCT
InversePCR 3	AGGCGTTTTATGTCAGAAGGCATTTAGAAT
TAIL AD1†	NGTCGASWGANAWGAA
TAIL AD2†	TGWGNAGSANCASAGA
TAIL AD3†	AGWGNAGWANCAWAGG
TAIL AD6†	WGTGNAGWANCANAGA
Splinkerette PCR adaptor and primers	
SPLK A	GAAGAGTAACCGTTGCTAGGAGAGACCGTGGCTGAATGAGACTGGTGTCGA CACTAGTGG
SPLK B Spe I Xba I	CTAGCCACTAGTGTCGACACCAGTCTCTAATTTTTTTTTTCAAAAAAA
SPLKFwd _1	GAAGAGTAACCGTTGCTAGGAGAGACC
SPLKFwd _2	GTGGCTGAATGAGACTGGTGTCGAC

Table S4. Oligonucleotides used in this study (continued)

Oligonucleotide name	Sequence
Inverse splinkerette PCR adaptor	
Sfi I_SPLK-A_GGG	ACTAGTGTCGACACCAGTCTCTAATTTTTTTTTTCAAAAAAA
Sfi I_SPLK-B	TATAGCTGGTCGTCGATTTCCTAACCTTCA
Primers for generating double strand lox66	
lox66_S	ACCGATAACTTCGTATAGCATACATTATACGAACGGTAGACGT
lox66_AS	CTACCGTTCGTATAATGTATGCTATACGAAGTTAT
Primers for sequencing recombined loxP and lox66/71 sites	
pRS400_U_primer_AS	GTGCACTCTCAGTACAATCT
AmpR_3'_AS	TTACCAATGCTTAATCAGTGAGGCACC
Other primers to determine insertion mutation sites	
BarcodePCR(888r)	CACGACATGTGCAGAGATGCCGACGAAGCA
InvU4 1366F	GTGCCAGGCGAGGGTATTATACAAGGCCTC
Ura4_EcoR V	TATAGCTGGTCGTCGATTTCCTAACCTTCA
Ade7_5'S	CTCCTGTTATCACGAAGCATGAAGAGT
Ade7_3'AS	GGTCAACCAGTTTCGAAGATATTGCTTATC
SPCA167.07c_'5S	AACAAGCTAACGGTTATGCCCAGCCGTT
SPAC167.97c_3'AS	CACGACATTGTCTAGATAGCCTTGC
SPCC1442.04c_5'S	AGATTCCACAAGTGAAGCCGAAACCCGA
SPCC1442.04c_3'AS	GATTCCTCAAGGTCGTTATCCCGCAAT
clg1 del 5'	AGAACCTGCGCACAACCAACCACCGTAAAATT
clg1 del 3'	GAAGAGGGCGATGGTGGTGCGTTGGTGCT
P12-SD-08-4031AS	AAGAAAATGAATACACAGAGTCAGAGAAAGAGATAGAAGGA
Brcd G1-08-4033	TGCTATCCCTGTACTTTTCCTTATCCTTTTTCCTTTGGG
SPAC24B11.08, 09 5' del	ACACAGGATCCGGCAACTTGTGCGATTTG
SPAC24B11.08, 09 3' del	AGCTAGCTTTTATTTGAAGATTAGGATGGCGT

† N = A, T, C or G; W = A or T; S = G or C
